# Understanding the burden of cognitive impairment associated with schizophrenia: Results from the international LUCIA study

**DOI:** 10.1192/j.eurpsy.2026.12208

**Published:** 2026-04-28

**Authors:** Christoph U. Correll, Silvana Galderisi, Maite Artés, Ana Fernández, Bregt Kappelhoff, Walter D. Lawhorn, Sébastien Tulliez, Satoru Ikezawa, Åsa Konradsson-Geuken, Morgane Sheykhi Hagaieg, Kari Skau, Monique van der Weijden-Germann, Robert A. McCutcheon

**Affiliations:** 1 Donald and Barbara Zucker School of Medicine at Hofstra/Northwell, USA; 2 Northwell, New Hyde Park, NY, USA; 3 Department of Child and Adolescent Psychiatry, Universitätsmedizin Charité Berlin, Berlin, Germany; 4 German Center for Mental Health (DZPG), Partner Site Berlin, Berlin, Germany; 5 German Center for Child and Adolescent Health (DZKJ), Partner Site Berlin, Berlin, Germany; 6 Einstein Center for Population Diversity (ECPD), Berlin, Germany; 7 University of Campania Luigi Vanvitelli, Naples, Italy; 8 Adelphi Targis S. L., Barcelona, Spain; 9 Boehringer Ingelheim B.V., Amsterdam, Netherlands; 10 Boehringer Ingelheim Pharmaceuticals Inc., Ridgefield, CT, USA; 11 Boehringer Ingelheim International GmbH, Ingelheim am Rhein, Germany; 12 National Center of Neurology and Psychiatry, Kodaira, Tokyo, Japan; 13 Department of Pharmaceutical Biosciences, Uppsala University, Uppsala, Sweden; 14 European Federation of Associations of Families of People with Mental Illness (EUFAMI), Leuven, Belgium; 15 Swedish Schizophrenia Association, Stockholm, Sweden; 16 Mobile Psychiatric Team RUBI, Saint-Étienne, France; 17 ACT-Team Moss, Hospital Østfold, Sykehuset Ostfold HF, Norway; 18 Psychologie praktijk van der Weijden-Germann, Groningen, The Netherlands; 19 Department of Psychiatry, University of Oxford, Oxford Health NHS Foundation, UK; 20 Oxford Health NHS Foundation Trust, Oxford, UK; 21 Department of Psychosis Studies, Institute of Psychiatry, Psychology and Neuroscience, King’s College, London, UK

**Keywords:** burden, CIAS, cognitive impairment, Delphi, schizophrenia

## Abstract

**Background:**

Cognitive impairment associated with schizophrenia (CIAS) is a prevalent, meaningful feature of schizophrenia with limited real-world data on its recognition and care setting impact. The LUCIA initiative is an international multi-stakeholder study that explored awareness, assessment practices, and the burden of CIAS to inform future care pathways.

**Methods:**

A three phase, Delphi-informed design was applied, comprising expert interviews to frame the enquiry, qualitative interviews with health and social care professionals (HCPs; n = 74) and caregiver advocates (n = 11), two waves of a Delphi survey among HCPs (n=449 and 343, respectively) and one round among 61 patients and 112 caregivers across 15 countries (n = 964).

**Results:**

The results showed poor awareness of CIAS across stakeholders. Structured cognitive assessment was infrequent, and clinicians largely relied on the dementia oriented Mini-Mental State Examination (MMSE) rather than schizophrenia specific tools, citing time, training, and unclear actionability as key barriers. CIAS imposed broad humanistic, clinical, societal, and economic burden – poorer quality of life, social isolation, higher comorbidities, increased hospital days and health care costs, and heavy informal care. Consensus actions prioritized the development of brief, validated screening instruments, improved psychoeducation, and accelerated research into effective pharmacological and non pharmacological interventions.

**Conclusions:**

These results provide additional evidence for the under-recognition of CIAS worldwide, despite its substantial multidimensional societal burden. The use of dementia-oriented cognitive tests carries significant risks of misclassification and inappropriate management. Therefore, improving awareness, implementing assessment guidelines, and accelerating therapeutic innovation is critical to improve the quality of life of CIAS patients and the wider community.

## Introduction

Cognitive impairment associated with schizophrenia (CIAS) is a core and enduring feature of schizophrenia. It affects multiple cognitive domains, including processing speed, attention/vigilance, working memory, verbal learning and memory, visual learning and memory, reasoning and problem-solving, and social cognition [1–4]. Considering the wide range of impairment severity from mild to severe, prevalence estimates are consistently high. In fact, between roughly 60 and 98% of individuals with schizophrenia meet the criteria for clinically relevant impairment [2, 5–7]. In line with the synaptic pruning theory of schizophrenia, research indicates that CIAS is often present in the prodromal phase of the disease, thus preceding the first psychotic episode [2, 8]. Moreover, CIAS persists throughout all subsequent phases of schizophrenia, highlighting the need for early identification and intervention to prevent long-term disability [2].

CIAS is a clinically meaningful aspect of schizophrenia that significantly influences everyday functioning and overall quality of life of patients and their caregivers. In fact, CIAS is a principal determinant of real‑world outcomes in schizophrenia and often outweighs positive symptom severity in predicting everyday functioning [9–11]. For example, deficits in verbal learning and memory, processing speed, and executive reasoning have consistently been associated with reduced quality of life, educational attainment, employment, and independent living [5, 12, 13]. In addition, deficits in social cognition, such as impaired emotion recognition and mentalizing, undermine the formation and sustaining of meaningful social relationships and limit community participation, compounding disability even when psychosis is otherwise controlled [14–16].

The negative functional impacts of CIAS also translate into a significant societal and economic burden. For example, patients with CIAS show higher health care utilization and cost compared to cognitively intact patients [17, 18]. Nevertheless, contemporary epidemiological and economic evidence remains heavily weighted toward claims-database studies from single health systems, leaving a fragmented picture of how CIAS is experienced across diverse healthcare, social-care, and cultural contexts.

Another key aspect of CIAS is how it is currently identified and monitored in the clinics. A wide array of clinical outcome assessment tools to measure CIAS are available, spanning comprehensive batteries, brief screening instruments, and interview‑based functional capacity measures [19, 20]. The MATRICS Consensus Cognitive Battery (MCCB) is the standard multidomain cognitive assessment for schizophrenia, endorsed by the European Psychiatric Association (EPA) and American Psychiatric Association (APA), due to its comprehensive domain coverage, strong reliability, and extensive clinical trial use [21–23]. Alternatively, brief cognitive tests and interview-based measures were developed for rapid CIAS screening and monitoring, overcoming the MCCB’s lengthy administration time (about 60 minutes) at the cost of reduced sensitivity and specificity [20, 24, 25]. Nevertheless, despite the availability of valid guideline-recommended tools, their use in routine clinical practice remains limited, as evidenced by prior research [21, 26]. To elucidate the underlying factors contributing to this implementation gap, there is a pressing need for pragmatic, multi-stakeholder data on real-world applications.

Despite compelling evidence that cognition is central to real‑world functioning and the availability of assessment tools, awareness and systematic management of CIAS across stakeholders remains suboptimal. Patients and caregivers frequently recognize day‑to‑day cognitive difficulties yet may not report them in clinical encounters, while clinicians cite time, training, and unclear actionability as barriers to structured assessment [10, 26, 27]. Concurrently, there is no approved pharmacological treatment for CIAS. Evidence regarding the cognitive effects of antipsychotics is mixed, augmentation strategies show inconsistent benefit, and although cognitive remediation therapy (CRT) demonstrates the strongest non‑pharmacological efficacy, access and fidelity vary widely [10, 28–32]. These realities heighten the importance of optimizing current practices and fostering the development of novel therapeutics.

To generate a better understanding of CIAS and identify current unmet needs, the objective of the “deLphi to Understand the burden of Cognitive Impairment Associated with schizophrenia” (LUCIA) project was to describe international stakeholders’ perspectives, including health and social care professionals (HCPs), patients, and caregivers, on the awareness, management, and humanistic, social, and healthcare burden of CIAS. Specifically, we aimed to: (i) quantify awareness and understanding of CIAS; (ii) characterize assessment practices and instrument use in routine care; (iii) delineate the clinical, humanistic, societal, and economic burden; and (iv) identify barriers and pragmatic opportunities to improve recognition and monitoring of CIAS, to ultimately inform guideline implementation and readiness for emerging treatments, and to improve the care pathway for people living with schizophrenia and their families.

## Methods

### Study design and rationale

We conducted a cross‑sectional, multi‑country, multi‑stakeholder study using a three‑phase, Delphi‑informed design to collate expert and experiential perspectives on CIAS. The design combined exploratory qualitative enquiry with iterative, standardized rating to support transparent consensus building where appropriate, and descriptive mapping where consensus was not the objective. Reporting follows the consensus‑method ACCORD guidelines, with essential methodological elements retained here and operational details available as Supplementary Methods [33, 34].

### Phase 1: Expert opinion

In‑depth individual interviews (IDIs) were conducted with 11 members of the Scientific Committee (SC), a heterogeneous group of experts from the participating countries and different relevant professional backgrounds involved in the management of schizophrenia. These interviews aimed to establish the contextual framework, refine the conceptual scope, and generate candidate domains and statements for subsequent enquiry. Insights from this stage informed the sampling frame for later phases and the architecture of the Phase 2 discussion guide and Phase 3 survey instruments.

### Phase 2: Qualitative enquiry

Semi‑structured, remote interviews (≈60 minutes) with 74 HCPs and 11 representatives of caregiver advocacy groups (CAGs) across 12 countries (multiple European countries plus China and Japan) to explore the patient journey and health‑care resource use, humanistic, economic and societal burden, and CIAS management. Thematic outputs informed item wording and prioritization in the Delphi survey.

### Phase 3: Quantitative Delphi survey

An online survey was deployed to HCPs, patients, and caregivers across 15 countries (multiple European countries plus China and Japan) to further explore the awareness of CIAS, patients’ experiences, and healthcare resources use, and the humanistic, economic, and societal burden of the condition. Two tailored versions of the survey were developed for HCPs and patients/caregivers, respectively. Items included single‑choice, multiple‑choice, open‑ended responses, and consensus statements rated on a 9‑point Likert‑type scale. Prior to full implementation, a soft launch was conducted to pilot the questionnaire, ensuring its clarity, comprehensibility, and operational functionality. The questionnaire was translated into local languages. Additional information on the methodology, including the definition of what is understood by a patient with or without CIAS, was included in the HCP questionnaire, and this is provided in the supplementary methodology material. The complete versions of the questionnaires are also provided as Supplementary Materials.

### Participants and eligibility

Panels included HCPs (psychiatrists, psychologists/psychotherapists, specialist nurses, social workers/occupational therapists, and hospital pharmacists), adult patients with schizophrenia, and caregivers/relatives. Countries spanned multiple European settings as well as China and Japan. HCP inclusion required documented experience managing schizophrenia and a minimum caseload in the prior year, with sampling targeted diversity of roles, sectors, and care settings. Patients and caregivers were recruited through a patient advocacy group (PAG). Full eligibility thresholds and sampling quotas are provided in the Supplementary Methods.

### Outcomes and consensus definition

The survey explored awareness of CIAS, patient pathways, and health‑care resource utilization, humanistic/economic/societal burden, treatment patterns, and unmet needs. For interpretive parsimony, Likert responses were grouped as disagreement (1–3), neutral (4–6), or agreement (7–9). A priori, consensus (agreement or disagreement) was defined as ≥70% of respondents selecting values within the same category. Items not reaching this threshold in wave 1 were reconsidered in wave 2 (HCPs only).

### Analysis

Analyses were primarily descriptive. Categorical variables are presented as frequencies/percentages, while continuous variables are presented as summary statistics. For consensus statements, category distributions and attainment of the ≥70% threshold were tabulated. Limited, pre‑specified exploratory comparisons (e.g., stratification by presence/absence of CIAS) were undertaken using Student’s *t*-test or chi-square as adequate, to avoid excess multiplicity. Data analysis was performed using IBM SPSS Statistics V.29.

### Ethical considerations

All participants provided informed consent prior to participation. Procedures were consistent with ethical principles for research involving human participants and applicable data‑protection requirements in participating jurisdictions. Formal ethics‑committee review was not required because this was an opinion-based survey without access to clinical records. Additional administrative details appear in the Supplementary Methods.

## Results

### Sample demographics and CIAS symptoms

**Figure 1.**
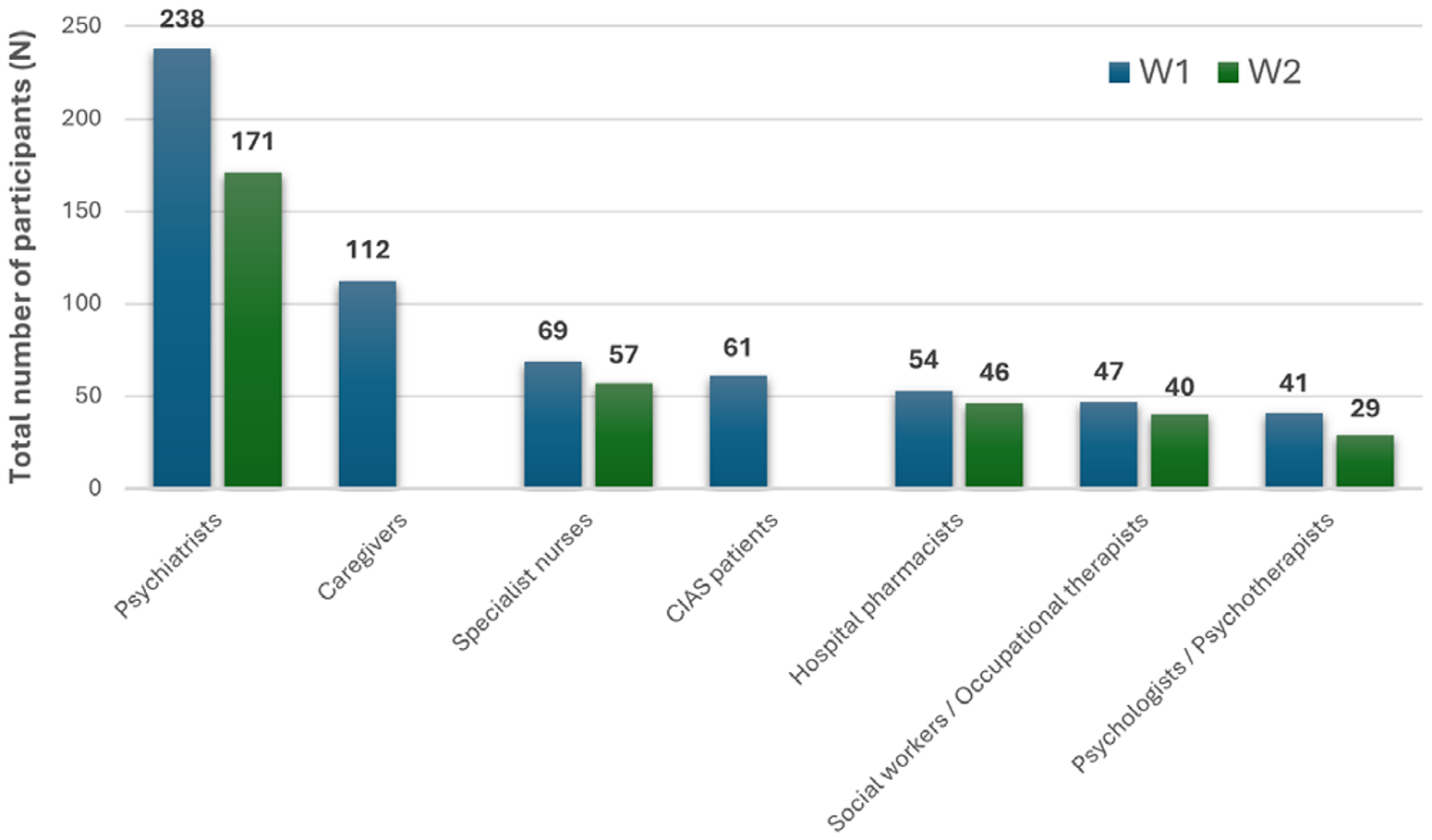
Delphi survey participation by target type across both Wave 1 (W1) and Wave 2 (W2), expressed as a total number of participants (*N*). CIAS, cognitive impairment associated with schizophrenia; W1, Wave 1; W2, Wave 2.

**Figure 2.**
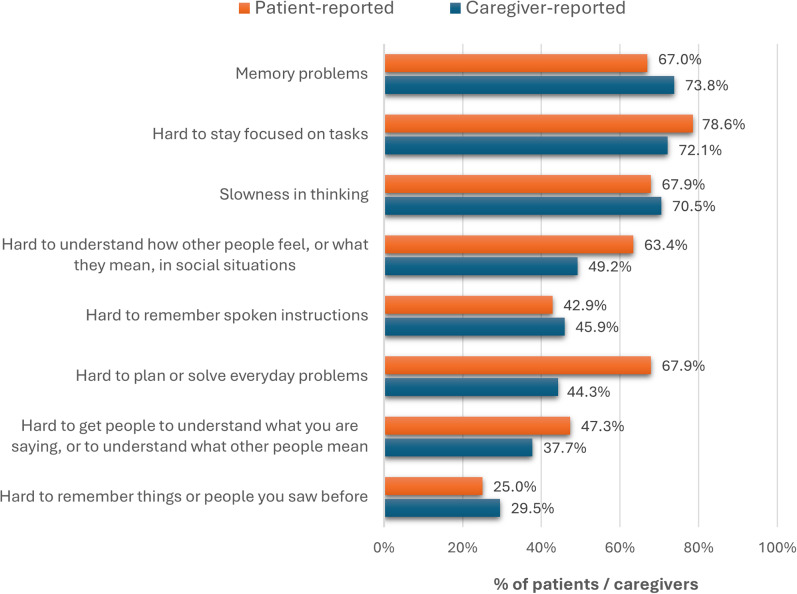
Prevalence of cognitive symptoms as reported by patients and caregivers, expressed as the percentage of total respondents (%).

A total of 61 patients and 112 caregivers completed the one‑shot survey, and 449 and 343 HCPs completed Delphi wave 1 (W1) and wave 2 (W2), respectively (Figure 1). Across all HCP specialties, most participants reported extensive experience managing schizophrenia (Table 1). Full patient and caregiver demographics, HCP demographics, and country‑level distributions are presented in Table 1 and Figure 1 of the Supplementary Results, respectively. Symptoms consistent with CIAS were frequently endorsed by both patients and caregivers: memory problems (67.0 and 73.8%, respectively), attention deficits (78.6 and 72.1%), and slowness in thinking (67.9 and 70.5%) (Figure 2).

**Table 1.** Demographic profile of health and social care professional panelists

Targets	Psychiatrists (*N* = 238)	Psychologists/ psychotherapists (*N* = 41)	Social workers/occupational therapists (*N* = 47)	Specialist nurses(*N* = 69)	Hospital pharmacists (*N* = 54)
Age (mean, SD)	48.1 (9.2)	47.0 (10.2)	39.7 (9.5)	41.1 (9.0)	44.6 (9.9)
Trained in psychology/psychotherapy (%)	84.0%	N/A	N/A	N/A	N/A
Type of working center (%)					
Public	52.1%	53.7%	48.9%	76.8%	81.4%
Private	18.5%	21.9%	42.6%	13.0%	9.3%
Both	29.0%	24.4%	4.3%	5.8%	9.3%
Other	0.4%	0%	4.3%	4.3%	0%
Type of practice (%)					
Teaching hospital: mental health	34.0%	31.7%	23.4%	34.8%	N/A
Non-teaching hospital: mental health	27.3%	17.1%	12.8%	24.6%	N/A
Public outpatient clinic	12.2%	12.2%	2.1%	2.9%	N/A
Private outpatient clinic	7.6%	2.4%	17.0%	2.9%	N/A
Primary care center	6.7%	12.2%	N/A	8.7%	N/A
Office	6.3%	9.8%	N/A	0%	0%
Community health-center/service	2.9%	7.3%	17.0%	1.4%	N/A
Teaching hospital: emergency	0.4%	2.4%	N/A	13.0%	72.2%
Non-teaching hospital: emergency	N/A	N/A	N/A	N/A	27.8%
Community social service	N/A	N/A	12.8%	N/A	N/A
Non-profit organization	N/A	N/A	6.4%	N/A	N/A
Other	1.7%	4.9%	8.5%	5.8%	0%
Working with SCZ patients for >15 years	54.6%	41.5%	14.9%(59.6% from 5 to 10 y)	46.4%	44.4%
Average number of patients per year (Mean, SD)	200 (213.6)	163.0 (184.0)	76 (79.3)	118.3 (139.5)	128.5 (121.9)

*Abbreviations:* N, number; N/A, not applicable; SD, standard deviation; y, years.

### Awareness and understanding of CIAS

Results from the one-shot survey showed that only 55.7% of patients and 65.2% of caregivers had heard the term “CIAS” before, confirming previous expert reports from the qualitative phase interviews. Similarly, Delphi results showed that only 52.2% of psychiatrists agreed that HCPs have a good understanding of how cognitive symptoms manifest, and 31.5% completely agreed that HCPs are not sufficiently aware that CIAS is part of schizophrenia.

Taken together, these findings suggest that routine psychoeducation regarding CIAS may be inconsistent and heterogeneous, as demonstrated by a lack of agreement on statements that HCPs routinely explain CIAS or warn patients that they may develop cognitive symptoms. Most stakeholders endorsed increasing CIAS awareness among psychiatric and non‑psychiatric HCPs, relatives and caregivers, and the wider society. Additional results, such as the reported reasons for lack of psychoeducation, main CIAS information sources, and agreement data on raising CIAS awareness, are available as Supplementary Results.

### Assessment practice and instrument use

Under-recognition of CIAS reported in qualitative interviews may partially explain the limited use of systematic clinical assessment practices. Results from the Delphi survey confirm the previous qualitative observations that CIAS is not routinely screened for, assessed, or monitored. In fact, only 15.8% of patients reported that HCPs often/very often assess cognition. Reported reasons mirror the SC findings – time constraints (64.6%), absence of protocols (53.9%), uncertain functional relevance (55.0%), lack of effective treatments (52.9%), and impractical tools (53.2%).

**Figure 3.**
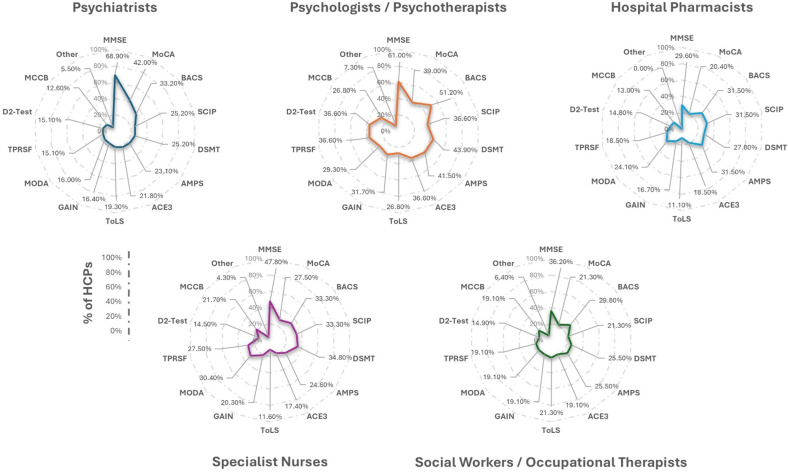
Frequency of utilization of CIAS assessment instruments by healthcare professionals (HCPs), represented as the percentage of total survey respondents. ACE3, Addenbrooke’s Cognitive Examination; AMPS, Assessment of Motor and Processing Skills; BACS, Brief Assessment for Cognition in Schizophrenia; CIS, Cognitive Impairment Scale; DSMT, Digit Span Memory Test; GAIN, Global Appraisal of Individual Needs; HCPs, healthcare practitioners; MCCB, MATRICS™ Consensus Cognitive Battery; MMSE, Mini Mental State Examination; MoCA, Montreal Cognitive Assessment; MODA, Memory Orientation and Dementia Assessment; SCIP, Screen for Cognitive Impairment; ToLS, Tower of London Scale; TPRSF, Test of Practical Judgment – Short Form.

In expert IDIs, tools utilized by HCPs to assess CIAS varied widely. Notably, the Mini‑Mental State Examination (MMSE) was most frequently used to screen (36.2%), identify (48.7%), and monitor (31.9%) CIAS; notably, 68.9% of psychiatrists reported using MMSE to *identify* CIAS (Figure 3). Usage of guideline concordant tools (e.g., MCCB, BACS) was comparatively lower and heterogeneous across professions. Experts reached consensus that new validated criteria to identify CIAS are needed (71.3%), whereas support for new screening criteria did not meet the consensus threshold (66.2%); there was disagreement about whether adequate validated criteria already exist for screening or diagnosis.

### CIAS follow-up

Qualitative interview results suggested a lack of clarity around roles and responsibilities for CIAS follow-up, which may hinder coordinated multidisciplinary management. Delphi results reinforced this idea since panelists reported limited social‑care follow‑up due to the absence of a freely available, validated monitoring scale, among other reasons (see Supplementary Results). Consensus (>70%) supported practical steps to strengthen monitoring: develop a short, validated tool for routine use; educate patients, families, and HCPs about CIAS; raise awareness via patient advocacy groups (PAGs); and deploy patient‑facing digital tools/apps to track cognitive symptoms.

### Disease burden

#### Humanistic burden

**Figure 4.**
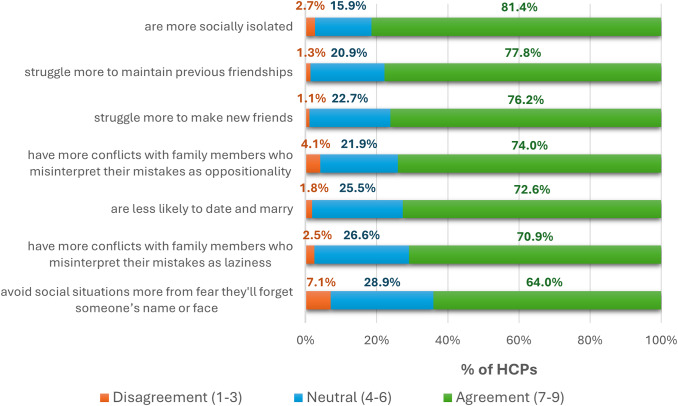
Expert-identified challenges among schizophrenia patients with and without cognitive impairment associated with schizophrenia (CIAS), reported as the percentage of total survey respondents (%). A priori, consensus (agreement or disagreement) was defined as ≥70% of respondents selecting values within the same category. HCPs, healthcare practitioners).

Qualitative interviews underscored the pervasive impact of CIAS on the patient’s identity, relationships, and well-being. In the survey, most patients endorsed the statements that cognition negatively affected their relationships (57.4%) and led to lost friendships (54.1%). Moreover, surveyed patients reported stigma/discrimination (35.8%) and difficulties starting a family (35.8%). According to HCPs, people with CIAS often felt poorly understood, leading to loneliness (62.3%) and hurt (52.5%). HCPs reached consensus that, compared with people with schizophrenia without CIAS, people with CIAS are more socially isolated, struggle to make and sustain friendships, are less likely to date/marry, and have more family conflict arising from misinterpreted cognitive errors (Figure 4).

#### Clinical burden

According to HCPs, CIAS was associated with higher utilization of acute and routine care. Compared with those without CIAS, patients with CIAS had longer routine psychiatry appointments (mean 29.5 vs 23.3 minutes; p < 0.01) and longer cognitive assessments (mean 29.9 vs 21.9 minutes; p < 0.01). They accrued more hospital days annually (mean 4.0 vs 2.9; p < 0.01), had longer inpatient stays (mean 32.4 vs 19.1 days; p < 0.01), and presented to the emergency department more often (mean 6.6 vs 4.7; p < 0.01). These data reflect greater clinical complexity, including relapse risk, comorbidity management, and safety concerns.

#### Comorbidities

**Figure 5.**
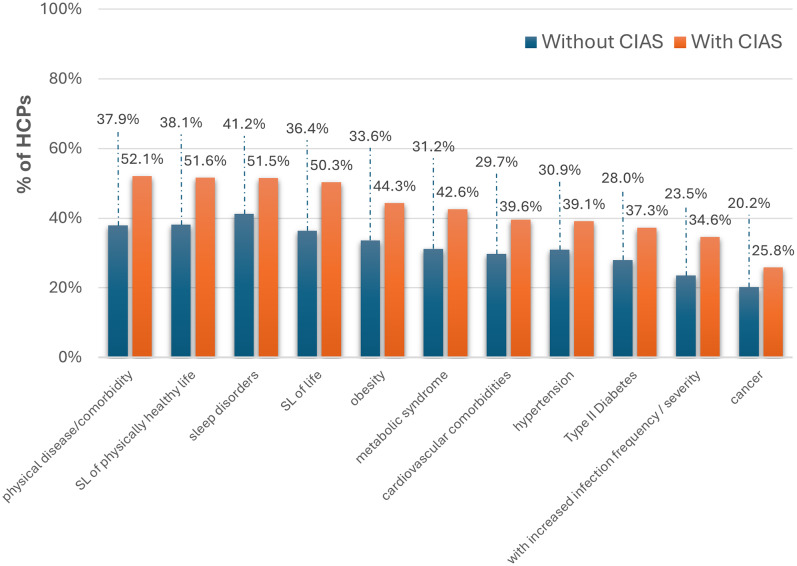
Physical morbidity among schizophrenia patients with and without CIAS, as reported by HCPs, expressed as a proportion of total survey respondents (%). CIAS,cognitive impairment associated with schizophrenia; SL, shortened length.

Compared with schizophrenia without CIAS, HCPs reported that patients with CIAS had higher rates of physical comorbidities (Figure 5). Experts agreed that CIAS is associated with shortened overall and healthy lifespan. The most common patient/caregiver-reported physical issue was sleep problems (49.7%). Reasons for elevated physical‑health burden were multifactorial and consistent with qualitative IDIs: missed early warning signs, forgetting medications, low physical activity, and communication challenges with clinicians (e.g., difficulty conveying symptoms or understanding advice).

**Figure 6.**
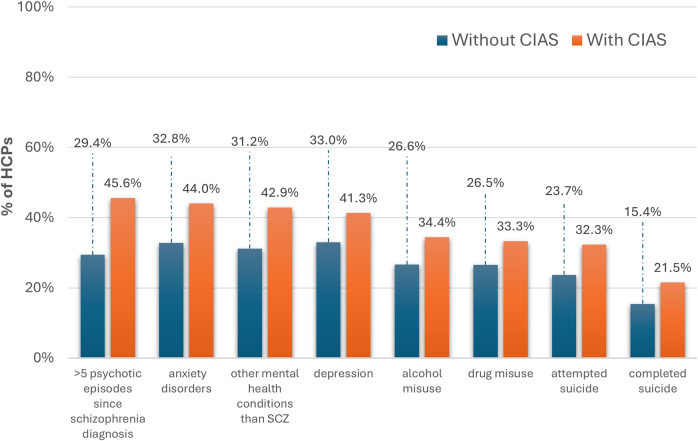
Prevalence of mental health comorbidities among individuals with schizophrenia, comparing those with and without CIAS (%) expressed by HCPs, presented as the proportion of total survey respondents. CIAS, cognitive impairment associated with schizophrenia HCPs, health-care practitioners.

Similar to physical comorbidities, high rates of mental comorbidities were reported by surveyed patients with schizophrenia, such as anxiety/panic disorders (52.0%), depression (35.8%), substance misuse (15.6%), and other mental disorders (15.0%). HCPs reached agreement (74.6%) that CIAS is associated with a higher incidence of other mental‑health conditions, including anxiety and depression, and that people with CIAS experience more psychotic episodes than those without CIAS. This claim aligns with HCP reports regarding mental health in patients with and without CIAS during the qualitative phases and insights shared by patients and caregivers during the Delphi phase (Figure 6). Although Delphi panels did not reach consensus on whether suicide risk is higher in CIAS, caregivers reported that patients had disclosed thoughts of ending their lives.

#### Societal burden

**Figure 7.**
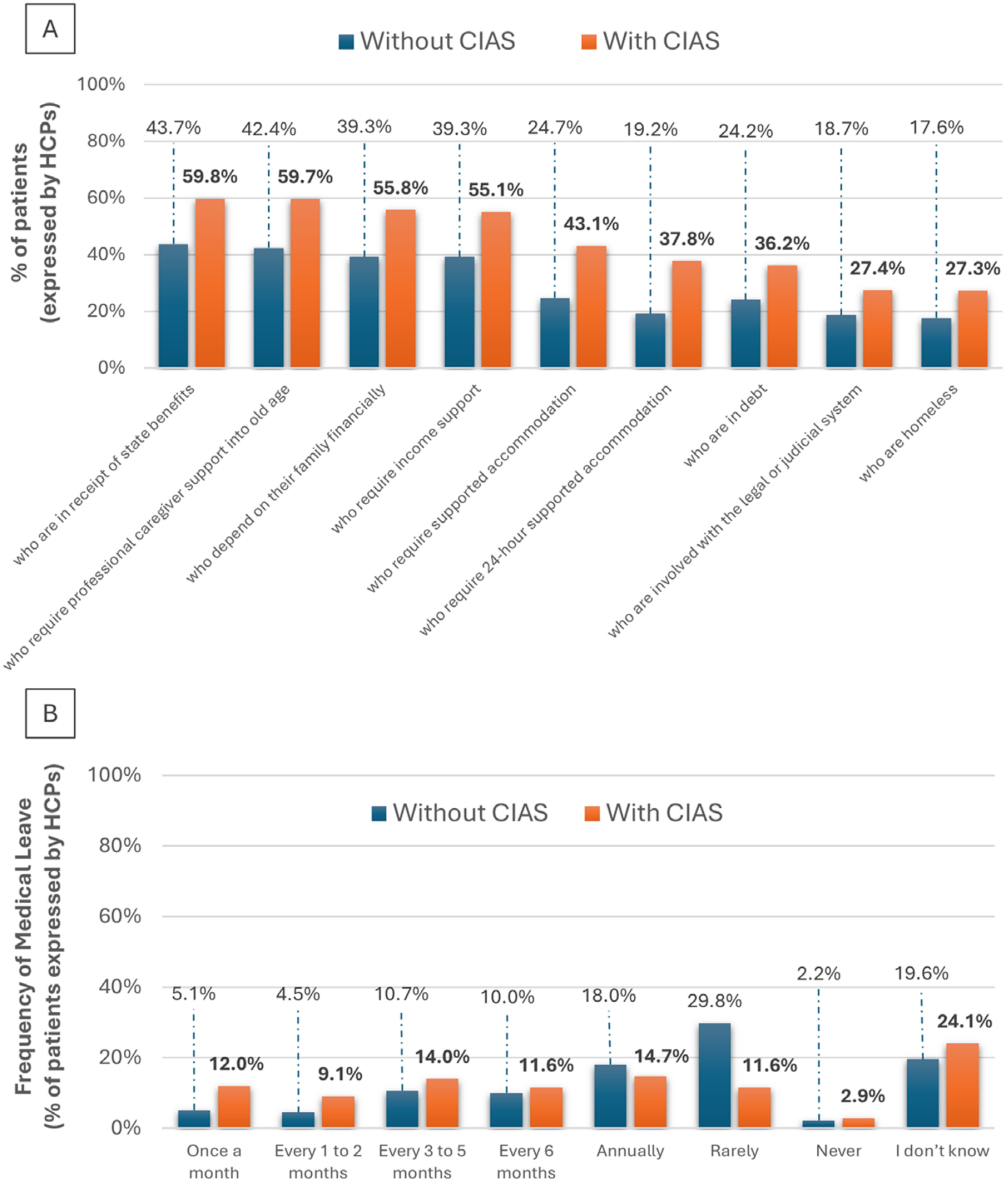
(A) Financial and social support status among individuals with schizophrenia, with and without CIAS, represented as the proportion of total survey respondents (%). (B) Frequency of medical leave from school or employment among individuals with schizophrenia, compared to those with and without cognitive impairment associated with schizophrenia (CIAS), presented as the proportion of total survey respondents (%). Abbreviations: CIAS, cognitive impairment associated with schizophrenia.

According to the surveyed patients, educational attainment and work participation in patients with schizophrenia are markedly constrained. Nearly half (47.5%) reported stopping school/education/work due to schizophrenia; 45.7% were unemployed, retired early because of sickness, or on long‑term sick leave. Workplace challenges were common: difficulty obtaining/retaining work (48.0%), moving to easier roles (50.8%), and insufficient day‑to‑day employer support (27.0%). Notably, as reflected in the responses of the HCPs on the Delphi panel, people with CIAS more often required medical leave from school or work than those without CIAS (Figure 7A). Beyond work and education, social resources were frequently needed, as shown by the patients’ survey: 15.0% struggled to keep a steady place to live; 32.5% lived with parents; 16.4% used supported accommodation; 14.8% received income support; and 26.2% received home‑based HCP support most or all the time. Relative to those without CIAS, people with CIAS were more likely to be in debt or homeless, engaged with the legal/judicial system, financially dependent on family, and to require supported accommodation (including 24‑hour support), state benefits, and professional caregiving into older age (Figure 7B).

#### Financial/economic burden

As expressed by HCP panelists, patients with CIAS cause a higher economic burden for society than patients without CIAS. Compared with schizophrenia without CIAS, the state spent significantly more on supported accommodation, home‑based social/medical support, and income‑replacement benefits (p < 0.001 for each; Table 2). Emergency department and inpatient costs were also higher (p < 0.001), mirroring elevated utilization. Overall, 42.5% of people with schizophrenia reported receiving financial assistance for medications.

**Table 2.** Comparison of health care resource utilization and associated costs between patients with schizophrenia with cognitive impairment (CIAS) and those without CIAS

Healthcare resource	Patients with CIAS *Mean* (SD)	Patients without CIAS *Mean* (SD)	p-value
Length of time per **routine visit to psychiatrist** (min)	29.5 (16.9)	23.3 (13.3)	<0.001
Time during the **visit to** the psychiatrist required to **assess cognition** (min)	29.9 (19.5)	21.9 (15.1)	<0.001
Money spent by the state/public health system on **routine psychiatry visits** per patient, per year (€)	2318.8 (9351.3)	1851.7 (5142.0)	0.372
Money spent by the state/public health system on **routine psychiatry visits to assess cognition** per patient, per year (€)	2307.0 (9581.7)	1276.3 (4618.3)	0.085
Number of **hospitalizations** per year	4.0 (8.4)	2.9 (8.3)	<0.001
Number of days of **hospital inpatient stay**	32.4 (28.9)	19.1 (16.1)	<0.001
Money spent by the state/public health system on **hospitalization costs** per patient, per year (€)	4564.6 (9171.2)	3418.2 (8148.0)	0.030
Money spent by the state/public health system on **hospital inpatient stays** per patient, per year (€)	5636.4 (11738.1)	3576.3 (7281.8)	0.003
Number of **presentations to the emergency department** per year	6.6 (11.8)	4.7 (10.0)	<0.001
Money spent by the state/public health system on **emergency department care** per patient, per year (€)	2585.8 (9352.0)	1744.4 (6205.7)	0.019
Money spent by the public health system on their **prescribed medications** per year (€)	2068.7 (3832.8)	1231.2 (2522.3)	<0.001
Money spent by the public health system on **non-pharmacological treatment** per year (€)	2102.5 (5018.5)	1454.3 (4516.0)	<0.001
Money spent by the patient on their **prescribed medications** per year (€)	1080.1 (3031.1)	681.1 (1863.4)	0.001
Money spent by the patient on **non-pharmacological treatment** per year (€)	739.8 (1492.9)	467.5 (1087.8)	<0.001
Money spent by the state/public health system on **supported accommodation** (€)	4759.2 (9898.1)	2683.8 (7641.3)	<0.001
Money spent by the state/public health system on **social support** at home (€)	2058.5 (3936.2)	1332.0 (4247.5)	0.001
Money spent by the state/public health system on **medical support at home** (€)	1520.1 (3613.1)	929.0 (2923.8)	<0.001
Money spent by the public health system on **state benefits** (€)	10532.2 (75270.5)	5746.2 (29320.8)	0.185

*Note:* Data are expressed as mean (± SD).

*Abbreviations:* SD, standard deviation.

#### Caregiver burden

**Figure 8.**
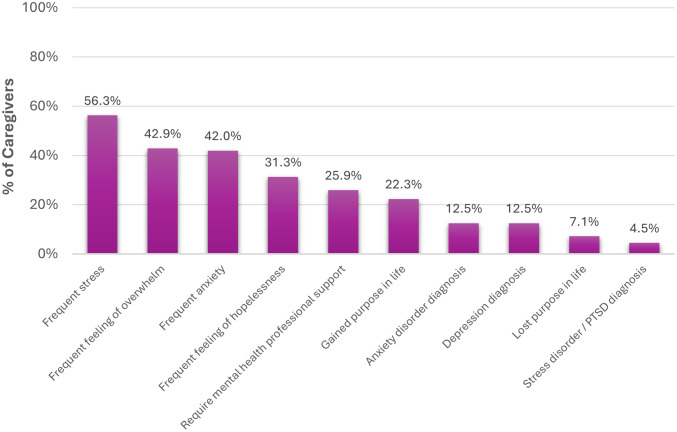
Mental health complaints reported by caregivers in relation to their caregiving responsibilities, presented as a proportion of total survey respondents (%). PTSD, post-traumatic stress disorder.

Surveyed caregivers reported providing extensive, largely informal support, on average 30.4 hours/week (SD 34.5), across instrumental and health‑navigation tasks (e.g., shopping, household management, prompting hygiene, medication reminders, scheduling and attending appointments, and completing formal applications). The economic opportunity cost is evident: only 37% were employed full‑time despite 52% holding a university degree, and 17.9% reported career slowing directly due to caregiving. Humanistic costs were pronounced: 49.1% did not socialize as much as they wished; 34.8% often felt lonely/isolated; and mental‑health impacts (e.g., stress) were frequent (Figure 8).

### Treatment landscape and unmet need

Consensus among HCP panelists was not reached on the effectiveness of either pharmacological or non‑pharmacological treatments for CIAS. Many HCPs reported considering switches to partial‑agonist antipsychotics (71.2%), though effectiveness did not meet consensus (66.7%). Similarly, there was no consensus regarding the use of methylphenidate or vortioxetine. Avoidance of anticholinergics due to secondary drug effects achieved agreement (71.8%), and most clinicians stated they apply this principle (71.2%).

For non‑pharmacological approaches, HCPs perceived effectiveness varied: Cognitive Behavioral Therapy 74.8%, CRT 65.3%, motivational interviewing 56.6%. Stakeholders rated sleep (85.1%), social rehabilitation/skills (82.4%), substance‑use interventions (82.2%), metabolic health (80.2%), occupational interventions (78.2%), and psychoeducation (73.5%) as particularly useful adjuncts. Looking forward, respondents endorsed more research to establish effective pharmacological (81.4%) and non‑pharmacological (80.0%) options, and 82.5% agreed that a new effective pharmacologic treatment for CIAS is needed. Despite the above results, 50.8% of patients reported never receiving treatment specifically for cognition.

**Figure 9.**
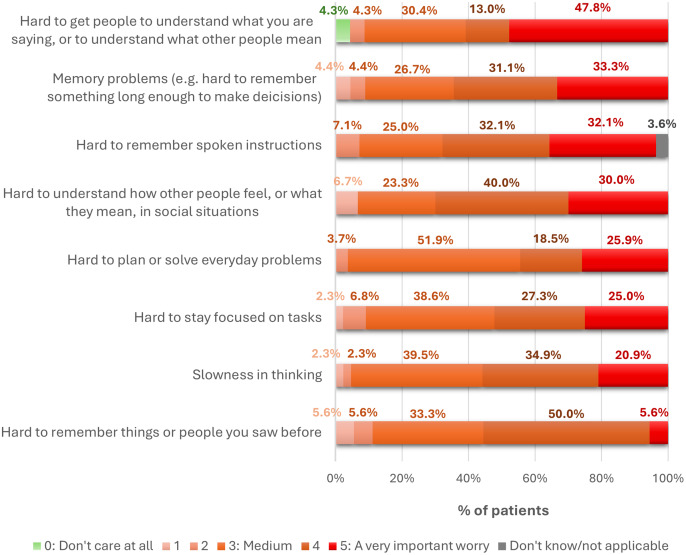
Patient-reported significance of individual cognitive symptoms, expressed as the proportion of total survey respondents (%).

**Figure 10.**
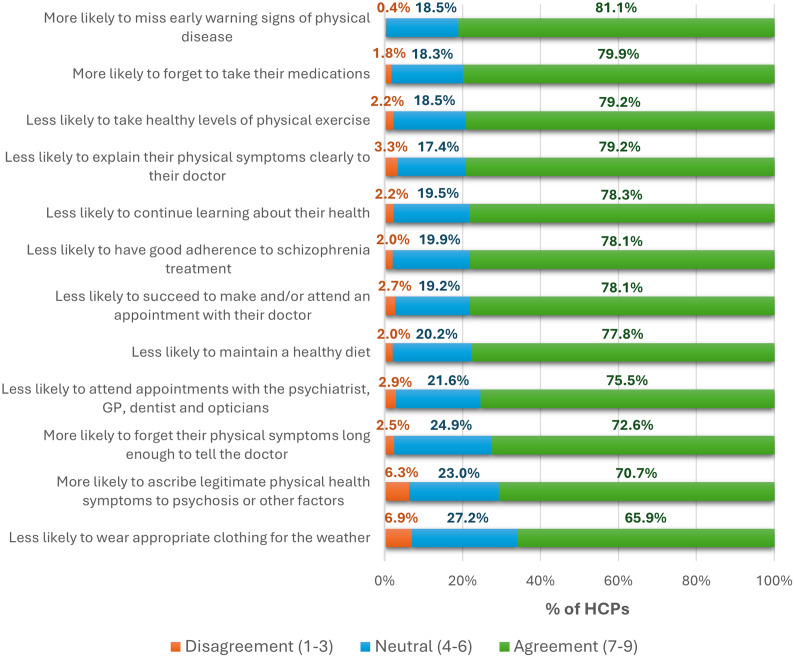
Expert-provided rationales for the elevated physical morbidity observed in schizophrenia patients with CIAS compared to those without CIAS. Data are presented as the proportion of total survey respondents for each item on the Likert scale (%). CIAS, cognitive impairment associated with schizophrenia; GP, general practitioner; HCPs, healthcare practitioner.

Patient-related concerns and behavioral mediators reveal unmet needs, thus providing potential targets for novel CIAS therapies. With regard to patient-reported concerns, communication and social‑understanding difficulties were among the most salient CIAS-related manifestations: 47.8% rated “difficulty getting others to understand what I say or understanding what others mean” as very important, followed by memory problems (33.3%) and “difficulties interpreting how others ‘feel’/”what they mean’ in social situations” (30.0%) (Figure 9). Qualitative data from SC IDIs highlighted neglect of personal health (e.g., missed GP appointments, forgetting to take medications), elevated substance use (alcohol, tobacco, drugs), and unhealthy lifestyle choices (poor diet, low exercise) as common among people with CIAS, compounding overall morbidity (Figure 10).

## Discussion

These findings reinforce the view that CIAS is a prevalent and clinically meaningful feature of schizophrenia, converging with extensive prior literature [1–3, 10]. The pattern hereby observed reflects substantial awareness of day‑to‑day cognitive difficulties among stakeholders, yet inconsistent formal recognition and documentation. This mirrors earlier reports that cognition is central to functional prognosis but remains under‑addressed in routine care [35–37]. Moreover, our findings align with previous studies showing negative impacts of CIAS on education, employment, social participation, and independent living [9, 12, 14, 15, 38].

Despite the centrality of CIAS, our data indicate that structured assessment remains uncommon and heterogeneous. Barriers described by participants, such as limited visit time, lack of training, unclear clinical pathways, costs/licensing, and perceived lack of actionability, are consistent with previously identified implementation hurdles [10, 19, 20]. A striking finding is the reliance on the dementia‑oriented MMSE by HCPs to identify CIAS. While familiarity and brevity likely drive this choice, the MMSE was neither designed nor validated for CIAS and is insensitive to mild to moderate CIAS profiles, potentially leading to false negative results [39, 40]. As a result, the use of inappropriate scales may distort care pathways, perpetuating the patient’s disability caused by CIAS. Therefore, it is of utmost importance for HCPs to adhere to current EPA and APA guidelines, recommending the MCCB as the reference multidomain assessment, and Screen for Cognitive Impairment (SCIP) and interview‑based measures as adjuncts for quick screening and contextualization [21, 22]. Despite their endorsement, however, these tools were relatively underutilized in our cohort, further demonstrating the enduring friction between psychometric optimality and clinical feasibility of assessment tools [20].

Despite the significant impact of CIAS on patients’ and caregivers’ lives, our findings reveal a considerable lack of awareness and formal recognition of CIAS among survey respondents. This gap underscores the need for improved education and structured approaches to CIAS assessment and management. Given psychiatrists’ role as primary clinical coordinators, education initiatives targeting psychiatric services are crucial to raise awareness of CIAS among patients and caregivers. However, education cannot rest solely on psychiatrists, whose workloads and time constraints already impede systematic assessment. Instead, multidisciplinary teams, including psychiatrists, specialist nurses, psychologists/psychotherapists, social workers/occupational therapists, and hospital pharmacists, should share responsibility for (i) opportunistic case‑finding; (ii) structured screening using brief, schizophrenia‑appropriate tools; and (iii) referral for comprehensive assessment where results are actionable [20, 21]. Moreover, patient advocacy groups (PAGs) can amplify psychoeducation for families, normalize discussions about cognitive health, and help bridge clinic‑to‑community gaps.

Our multi‑stakeholder data underscore the breadth of CIAS-related burden. Humanistic consequences, such as lower quality of life, reduced autonomy, and stigma, intersect with clinical complexity, including cardiometabolic comorbidities that may exacerbate cognitive deficits and treatment challenges [41]. Societal and economic costs are manifested in reduced educational attainment, unemployment/underemployment, greater need for supported living, and increased reliance on health‑care and social services [12, 14, 18, 38]. Translating these insights into action will require targeted social supports (e.g., programs enhancing social integration), vocational rehabilitation tailored to common cognitive profiles, and enhanced support for caregivers delivered in partnership with PAGs.

Despite the high burden of CIAS, our respondents highlighted a pervasive sense that individuals with CIAS are underserved. In fact, pharmacological options are limited: there is no approved treatment for CIAS, while augmentation strategies show inconsistent benefit [3, 28, 29, 32]. Non‑pharmacological interventions, most notably CRT, have the strongest evidence base, but access, fidelity, and funding are uneven [5, 30]. Minimizing anticholinergic burden, increasingly recognized as deleterious to cognition, represents a tangible short‑term optimization target [31]. These realities argue for coordinated strategies that prioritize research into targeted pharmacological therapies and rigorous evaluation of real‑world effectiveness for both pharmacological and non‑pharmacological approaches and invest in service capacity for CRT and related interventions (e.g., software and structured activities).

This cross-sectional opinion-based study has limitations of potential selection/recall biases. Also, Delphi processes can be influenced by item framing and by who participates. Instrument use and burden estimates may be sensitive to national service configurations, cultural norms, and reimbursement structures, limiting the generalizability of results.

## Conclusion

Despite the burden of CIAS, unmet needs remain widespread, spanning awareness, identification, and treatment. Therefore, urgent measures are required to close these gaps, including enhanced education, management strategies, and patient/caregiver support. By reshaping perceptions and enhancing service provision, it is possible to address the complex, layered challenges of CIAS, ultimately improving quality of life and outcomes for individuals and the wider community.

## Supporting information

10.1192/j.eurpsy.2026.12208.sm001 Correll et al. supplementary material Correll et al. supplementary material

## Data Availability

To ensure independent interpretation of clinical study results and to enable authors to fulfill their roles and obligations under the ICMJE criteria, Boehringer Ingelheim grants all external authors access to relevant clinical study data. In adherence with the Boehringer Ingelheim Policy on Transparency and Publication of Clinical Study Data, scientific and medical researchers can request access to clinical study data. Researchers should use the https://vivli.org/ link to request access to study data and visit https://www.mystudywindow.com/msw/datasharing for further information.

## Supplementary material

The supplementary material for this article can be found at http://doi.org/10.1192/j.eurpsy.2026.12208.
