# Supplementary material for: Understanding the burden of cognitive impairment associated with schizophrenia: Results from the international LUCIA study
Source: Eur Psychiatry. 2026 Apr 28;69(1):e55. doi: 10.1192/j.eurpsy.2026.12208 (PMC13227134; doi:10.1192/j.eurpsy.2026.12208)
Supplement: Correll et al. supplementary material [file S0924933826122081sup001.zip › SUPPL_MAT_1_HCP_Questionnaire.docx]

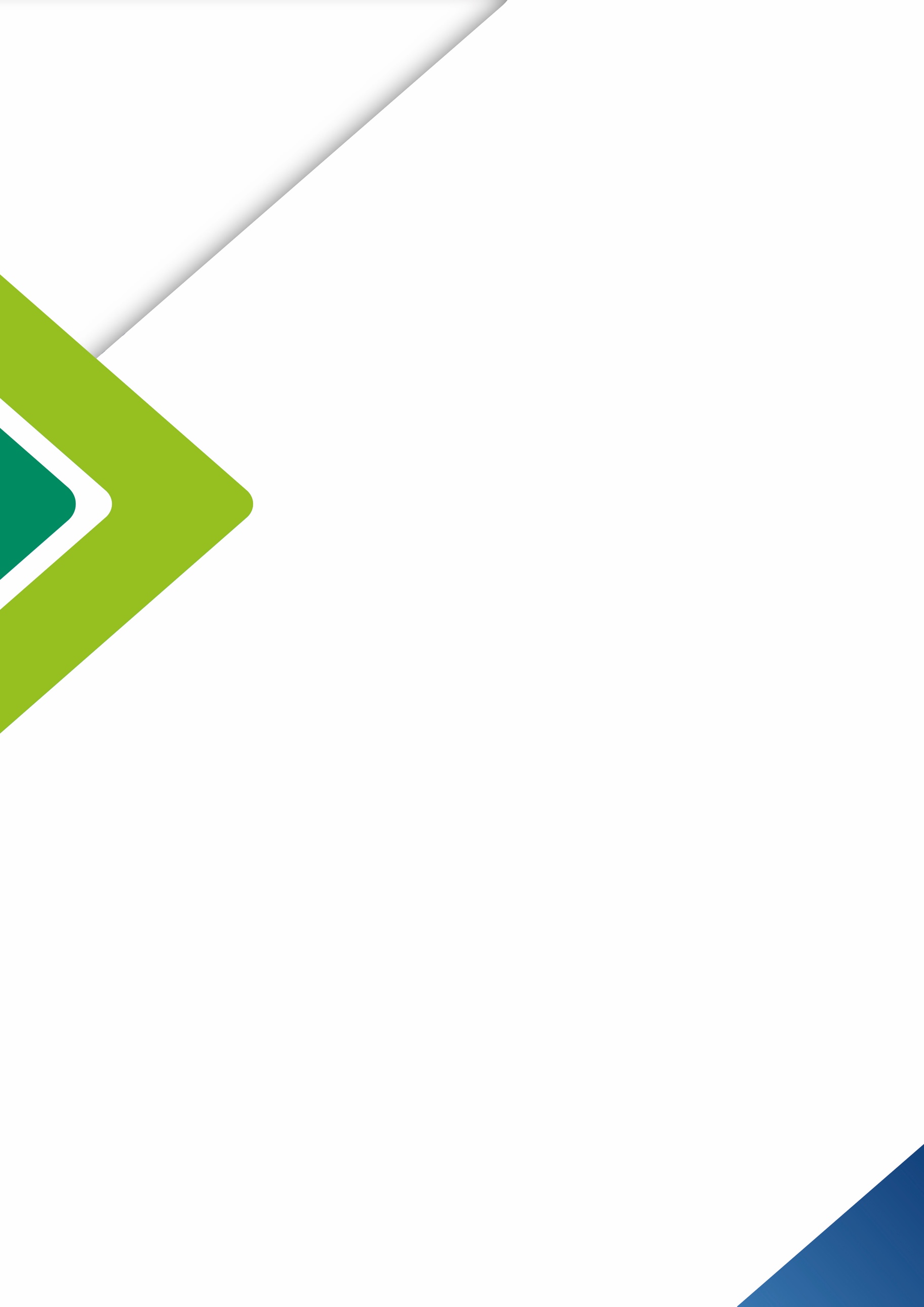


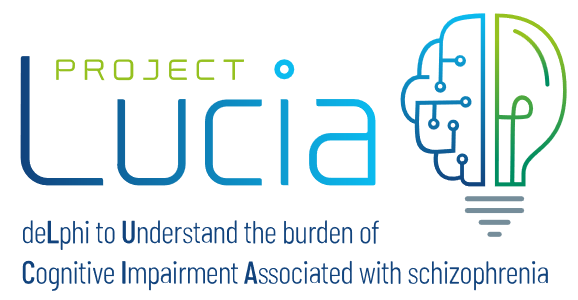


Delphi: HCP Questionnaire (Wave 1) Final Version

MAY 2024

# Disclaimer

Please note that the research design of this survey requires us to use persistent identifiers, in line with our privacy policy [LINK]. By applying persistent identifiers, Adelphi Targis is able to combine and review your responses to this and future follow-up surveys, as to get an in-depth longitudinal picture of your insights. In addition to this survey, you shall have the opportunity to complete other follow-up survey around similar topics, across a year. You are not obligated to participate in this or any follow-up surveys. You may withdraw or refuse any market research opportunities as you see fit.

[PROGRAMMING: THIS PARAGRAPH ONLY IN ENGLISH VERSION] This market research will be conducted in accordance with the MRS (Market Research Society) Guidelines, the BHBIA Legal & Ethical Framework and the ABPI Code of Conduct, as well as adhering to Data Protection legislation.

[PROGRAMMING: THIS PARAGRAPH ONLY IN SPANISH VERSION]: Nos gustaría asegurarle que actuamos de conformidad con todas las leyes y reglamentos pertinentes (los códigos de conducta ESOMAR y EphMRA relativos al anonimato y la confidencialidad.

Are you happy for Adelphi Targis to use persistent identifiers in this survey? Should you agree to participate, your responses to this and future follow-up surveys will be analysed in tandem. By clicking on the box below, you confirm to have read, understood and accepted these terms, and wishes to proceed with the market research survey on this basis:

1. I accept. (can participate and ID can be matched across waves - Mandatory)
2. I don’t accept. [PROGRAMMING: If selected, please show the following warning message: *Please read the information provided and select the ‘I accept’ option in order to continue with the survey. Otherwise, you will not be able to participate in this study. Thank you*.]

# Adverse Event

**In the event of an adverse event…**

We are required to pass on to our client details of adverse events/product complaints pertaining to their products that are mentioned during the study. If this happens, we will need to collect details and report the event, even if you have already done so personally.

You will be asked whether you consent to us passing your details to the client company’s drug safety department for their follow up, but you may choose to remain anonymous. This will have no impact on the confidentiality and anonymity associated with the study itself.

[PROGRAMMING: FOR UK ONLY]

We are required to pass on details of adverse events and product complaints to our client that are mentioned during the course of this market research. Although what you say will, of course, be treated with confidentiality, should you raise an adverse event or product complaint in a specific patient or group of patients during the discussion, we will need to report this even if it has already been reported by you directly to the company or the regulatory authorities using the MHRA's 'Yellow Card' system. In such a situation you will be asked whether or not you are willing to waive the confidentiality given to you under the Market Research Codes of conduct specifically in relation to that adverse event. Everything else you say during the course of the interview will continue to remain confidential, and you will still have the option to remain anonymous if you so wish.

Do you agree to participate with the interview on this basis?

1. I agree. (Mandatory)
2. I don’t agree. [PROGRAMMING: If selected, please show the following warning message: *Please read the information provided and select the ‘I agree’ option in order to continue with the survey. Otherwise, you will not be able to participate in this study. Thank you*.]

[PROGRAMMING: FOR GERMANY ONLY]

**Hinweis Meldepflicht**

Pharmaunternehmen sind gesetzlich dazu verpflichtet, Meldungen über Verdachtsfälle von Nebenwirkungen (unerwünschte Ereignisse), andere für die Patientensicherheit relevanten Informationen (wie z.B. Schwangerschaften, Medikationsfehler, etc.) und/oder Produktbeanstandungen zu erfassen und an Gesundheitsbehörden weltweit zu berichten.

Sollten Sie uns während der Befragung Informationen zu einer solchen Situation mitteilen, so sind wir verpflichtet, diese Information(en) mit allen uns bekannten Details an das pharmazeutische Unternehmen weiterzuleiten, selbst wenn Sie dieses bereits direkt dem Unternehmen oder den zuständigen Behörden berichtet haben. Diese Meldung erfolgt anonym, ohne Angaben zu Ihrer Person.

In manchen Fällen kann es sein, dass das pharmazeutische Unternehmen Rückfragen zu den berichteten Informationen hat. In diesen Fällen sind wir verpflichtet, zu versuchen diese offenen Rückfragen mit Ihnen zu klären und würden Sie ggf. hierfür kontaktieren

[PROGRAMMING: FOR SPAIN ONLY]

**Eventos adversos**

Aunque toda la información que nos facilite en esta encuesta se tratará de forma confidencial, si menciona algún efecto secundario de un medicamento o cualquier otra información de seguridad, por ejemplo, sobre la seguridad con la que se utiliza un medicamento, y/o una reclamación sobre la calidad de un medicamento durante la cumplimentación de este cuestionario, tendremos que comunicárselo a la empresa, aunque usted ya lo haya hecho.

En tal caso, se le preguntará si está dispuesto a renunciar a la confidencialidad que le otorgan los Códigos de Conducta de Investigación de Mercado específicamente en relación con ese efecto secundario u otra información de seguridad y/o queja sobre la calidad del medicamento. Esto nos permitirá informar y estudiar el problema más a fondo. Siempre tiene derecho a permanecer en el anonimato y, si decide no facilitar sus datos de contacto, solo se le identificará por su función. Cualquier otra información que facilite durante la encuesta será confidencial.

# Delphi Questionnaire: Introduction

Thank you very much for your interest in participating in the LUCIA Project: de**L**phi to **U**nderstand the burden of **C**ognitive **I**mpairment **A**ssociated with schizophrenia.

The ultimate objective of this project is to provide a multidisciplinary consensus on understanding the burden of Cognitive Impairment Associated with Schizophrenia (CIAS) among various professionals involved in its management.

The **DELPHI methodology** will be used, a commonly employed research technique in healthcare. This approach involves administering a questionnaire twice, referred to as the 1st and 2nd waves.

In this project, consensus shall be deemed to have been reached when 70% or more of the participants agree or disagree on the same statement. During the 2nd wave, questions for which a consensus was not reached in the 1st wave will be revisited.

The questionnaire is structured into **5 sections**:

- SECTION 1. Panellist Profile
- SECTION 2. Patient Pathway and HCR Use
- SECTION 3. Humanistic, Economic and Societal Burdens / Gaps
- SECTION 4: CIAS Treatment
- SECTION 5. CIAS Burden

**There are no right or wrong answers**, so please feel free to give your point of view and express your level of agreement or disagreement with the different statements based on your expertise and your professional opinion.

The platform allows you to save your progress, so you will be able to access the questionnaire as often as you need to complete it during this period.

**Once again, thank you for your time. Let’s begin!**

# Delphi Questionnaire

## SECTION 1: Panellist Profile

*Please answer the following questions, which are intended to characterise the participants and their fit into the inclusion criteria. All your answers will be kept confidential.*

1. **How old are you?** (Please enter your answer)

|__|__| years of age Range 0-99

1. **What is your primary job role?** (Please select one answer)

- Hospital Pharmacist CODE 1
- Nurse CODE 2
- Occupational Therapist CODE 3
- Psychiatrist CODE 4
- Psychologist / Psychotherapist CODE 5
- Social Worker CODE 6
- Other → Please, specify: _____________ (END)

1. [PROGRAMMING: this question is only for psychiatrists CODE 4 in Q2)] **Are you trained/sub-specialised in psychology or psychotherapy?** (Please select one answer)

- Yes
- No

1. **In which country is your practice based?** (Please select one answer)

- Belgium
- China
- Denmark
- Finland
- France
- Germany
- Greece
- Italy
- Japan
- Netherlands
- Norway
- Portugal
- Spain
- Sweden
- United Kingdom
- Other → Please, specify: _____________ (END)

1. **Do you work at a public, private or both types of centres?** (Please select one answer)

- Public centre/ practice
- Private centre/ practice
- Both
- Other → Please, specify: _____________

1. [PROGRAMMING: this question is only for psychologists/psychotherapists, psychiatrists and nurses CODE 2, 4 & 5 in Q2)] **What kind of hospital/centre do you work in (please, select where you spend the most time in your working day)?** (Please select one answer)

- Teaching hospital: mental health department
- Non-teaching hospital: mental health department
- Teaching hospital: emergency unit
- Non-teaching hospital: emergency unit
- Public outpatient clinic
- Private outpatient clinic
- Individual office/office shared with other healthcare professionals
- Primary care centre
- Community service
- Other → Please, specify: _____________

1. [PROGRAMMING: this question is only for pharmacists CODE 1 in Q2)] **What kind of hospital/centre do you work in (please, select where you spend the most time in your working day)?** (Please select one answer)

- Teaching hospital
- Non-teaching hospital
- Community Pharmacy (END)
- Individual office/office shared with other healthcare professionals (END)
- Other → Please, specify: _____________ (END)

1. [PROGRAMMING: this question is only for social workers CODE 6 in Q2)] **What kind of hospital/centre do you work in (please, select where you spend the most time in your working day)?** (Please select one answer)

- Community health centre or service
- Community social service
- Private practice
- Teaching hospital
- Non-teaching hospital
- Outpatient clinic
- Nursing home
- Non-profit organisation
- School or educational institution
- Other → Please, specify: _____________

1. [PROGRAMMING: this question is only for occupational therapists CODE 3 in Q2) ] **What kind of hospital/centre do you work in (please, select where you spend the most time in your working day)?** (Please select one answer)

- Teaching hospital
- Non-teaching hospital
- Outpatient psychiatry clinic
- Private practice
- Community health centre or service
- Community social service
- Other → Please, specify: _____________

1. **For how long have you been working with patients/clients with schizophrenia?** (Please select one answer)

- <5 years (END)
- 5-10 years
- 11-15 years
- >15 years

1. **How many people with schizophrenia have you personally seen, diagnosed, treated or followed up in the last 12 months?** (Please think of the number of patients, not visits per patient)

_____ patients in the last 12 months (Range 0-9999 / If < 50 🡪 END)

1. **What percentage of your patients/clients with schizophrenia experience cognitive symptoms due to their schizophrenia (i.e. not primarily due to another factor, e.g. age)?**

_____ % (Range 0-100 / If < 40% 🡪 END)

1. [PROGRAMMING: this question is only for psychologists/psychotherapists, psychiatrists, hospital pharmacists and nurses: CODE 1, 2, 4 and 5 in Q2)] **In your regular clinical practice, please indicate in which of the following phases of the schizophrenia patient/client journey you are involved:** (Please select one answer per column)

|  | **Initial assessment and referral** | **Diagnosis** | **Treatment** | **Follow-up** |
| --- | --- | --- | --- | --- |
| Yes | ÿ 1 | ÿ 3 | ÿ 5 | ÿ 7 |
| No | ÿ 2 | ÿ 4 | ÿ 6 | ÿ 8 |

1. [PROGRAMMING: this question is only for psychologists/psychotherapists and psychiatrists who select “Yes” for “Treatment”: CODE 5 in Q13)] **What type of treatment do you administer for cognitive symptoms associated with schizophrenia?** (Please select all that apply)

- Cognitive Remediation Therapy (CRT)
- Cognitive Behavioural Therapy (CBT)
- Eye Movement Desensitization Reprogramming (EMDR)
- Transcranial Magnetic Stimulation (TMS) / Transcranial Direct Current Stimulation (tDCS)
- Psychoeducation
- Psychoanalysis
- Motivational Interviewing
- Social Rehabilitation / Social Skills Training (e.g. encouraging voluntary work, social groups)
- Occupational Interventions
- Addressing Substance Use
- Addressing Sleep
- Addressing Metabolic Health / Physical Exercise
- Prescription of Pharmacological Agents
- Deprescription of Pharmacological Agents (i.e. those that are exacerbating cognitive symptoms)
- Other → Please, specify: _____________

1. [PROGRAMMING: this question is only for social workers and occupational therapists: CODE 3 in Q2)] **In your regular practice, please indicate in which of the following phases of the schizophrenia patient journey you are involved:** (Please select one answer per column)

|  | **Initial assessment** | **Diagnosis** | **Treatment, including treatment planning / coordination** | **Ongoing support / Follow-up** |
| --- | --- | --- | --- | --- |
| Yes | ÿ | ÿ | ÿ | ÿ |
| No | ÿ | ÿ | ÿ | ÿ |

1. **Based on your knowledge and experience, please state your degree of agreement with the following statements related to the awareness of cognitive symptoms associated with schizophrenia, being 1 completely disagreeing and 9 completely agreeing:** (Please select one answer per row)

| **Awareness of cognitive symptoms among people with schizophrenia** | **1**  **Completely**  **disagree** | **2** | **3** | **4** | **5** | **6** | **7** | **8** | **9**  **Completely**  **agree** | **I do not know / it does not apply to me** |
| --- | --- | --- | --- | --- | --- | --- | --- | --- | --- | --- |
| 1. Cognitive symptoms are a core feature of schizophrenia. | ÿ | ÿ | ÿ | ÿ | ÿ | ÿ | ÿ | ÿ | ÿ | ÿ |
| 1. Healthcare practitioners have a good understanding of how cognitive symptoms manifest in schizophrenia. | ÿ | ÿ | ÿ | ÿ | ÿ | ÿ | ÿ | ÿ | ÿ | ÿ |
| 1. Healthcare practitioners seeing patients with schizophrenia routinely tell them that they might have/get cognitive symptoms. | ÿ | ÿ | ÿ | ÿ | ÿ | ÿ | ÿ | ÿ | ÿ | ÿ |
| 1. Healthcare practitioners seeing patients with schizophrenia routinely screen for cognitive symptoms. | ÿ | ÿ | ÿ | ÿ | ÿ | ÿ | ÿ | ÿ | ÿ | ÿ |
| 1. Healthcare practitioners feel confident screening for cognitive symptoms in patients with schizophrenia. | ÿ | ÿ | ÿ | ÿ | ÿ | ÿ | ÿ | ÿ | ÿ | ÿ |
| 1. Healthcare practitioners seeing schizophrenic patients with cognitive symptoms routinely educate them about what cognitive symptoms mean (psychoeducation). | ÿ | ÿ | ÿ | ÿ | ÿ | ÿ | ÿ | ÿ | ÿ | ÿ |
| 1. Healthcare practitioners seeing schizophrenic patients with cognitive impairment routinely educate them about how to manage cognitive symptoms. | ÿ | ÿ | ÿ | ÿ | ÿ | ÿ | ÿ | ÿ | ÿ | ÿ |
| 1. Healthcare practitioners seeing schizophrenic patients with cognitive symptoms routinely educate family members about how to manage cognitive symptoms. | ÿ | ÿ | ÿ | ÿ | ÿ | ÿ | ÿ | ÿ | ÿ | ÿ |
| **Awareness of cognitive symptoms in patients with schizophrenia** | **1**  **Completely**  **disagree** | **2** | **3** | **4** | **5** | **6** | **7** | **8** | **9**  **Completely**  **agree** | **I do not know / it does not apply to me** |
| 1. People with schizophrenia and mild cognitive symptoms usually know they have poor cognitive health. (mild: able to maintain daily activities independently) | ÿ | ÿ | ÿ | ÿ | ÿ | ÿ | ÿ | ÿ | ÿ | ÿ |
| 1. People with schizophrenia and moderate-severe cognitive symptoms usually know they have poor cognitive health. (moderate-severe: require support to maintain daily activities) | ÿ | ÿ | ÿ | ÿ | ÿ | ÿ | ÿ | ÿ | ÿ | ÿ |
| 1. People with schizophrenia and cognitive symptoms often blame these cognitive symptoms on side effects from their psychotropic medications. | ÿ | ÿ | ÿ | ÿ | ÿ | ÿ | ÿ | ÿ | ÿ | ÿ |

## SECTION 2. Patient Pathway

*Please read the following text carefully, it is important for the next stage of questions…*

*Cognitive Impairment Associated with Schizophrenia (CIAS) affects the following domains, in a global manner:*

- *Attention/vigilance (e.g. struggle to read a book),*
- *Working memory (e.g. struggle to remember a phone number just given to you),*
- *Verbal learning and memory (e.g. remembering the items someone told you to buy at the supermarket),*
- *Visual learning and memory (e.g. remembering where you put something in a closet),*
- *Reasoning and problem solving (e.g. arriving on time for work even when the bus schedule has changed),*
- *Speed of processing (e.g. using a touch-screen computer to serve customers at a fast-food restaurant), and*
- *Social cognition (e.g. knowing by looking at someone whether they are angry at you or not).*

*Cognitive symptoms differ from the negative symptoms of schizophrenia which mainly include blunted affect, alogia (lack of speech), reduced motivation and anhedonia (reduced experience of pleasure).*

*CIAS also differs from dementia, which comprises symptoms that are often restricted to the domains of memory.*

*In this section, please answer the following questions to give us an understanding of CIAS. If there are certain items for which you don't have an opinion, kindly select 'I do not know / it does not apply to me.' Please use this option only when you genuinely lack an opinion on the matter. Your honest responses are highly valued. Thank you!*

1. **Based on your knowledge and experience, are males more likely to have CIAS than females?**

- Yes
- No
- I do not know

1. **Based on your knowledge and experience, please state your degree of agreement with the following statements related to mentioning cognitive symptoms to people with schizophrenia, being 1 completely disagreeing and 9 completely agreeing:** (Please select one answer per row)

| Mentioning cognitive symptoms to people with schizophrenia | **1**  **Completely**  **disagree** | **2** | **3** | **4** | **5** | **6** | **7** | **8** | **9**  **Completely**  **agree** | **I do not know / it does not apply to me** |
| --- | --- | --- | --- | --- | --- | --- | --- | --- | --- | --- |
| 1. Some healthcare practitioners treating people with schizophrenia inform these patients about the possibility of having/getting cognitive symptoms. | ÿ | ÿ | ÿ | ÿ | ÿ | ÿ | ÿ | ÿ | ÿ | ÿ |
| There may be different reasons why healthcare practitioners do not tell their patients with schizophrenia about cognitive symptoms.  In your view, the underlying reason might be... |  |  |  |  |  |  |  |  |  |  |
| 1. …they don’t want to make their patients afraid. | ÿ | ÿ | ÿ | ÿ | ÿ | ÿ | ÿ | ÿ | ÿ | ÿ |
| 1. …they don’t want to make their patient’s family members afraid. | ÿ | ÿ | ÿ | ÿ | ÿ | ÿ | ÿ | ÿ | ÿ | ÿ |
| 1. …because there is no effective pharmacological treatment for CIAS. | ÿ | ÿ | ÿ | ÿ | ÿ | ÿ | ÿ | ÿ | ÿ | ÿ |
| 1. ....because treatment for CIAS is not funded. | ÿ | ÿ | ÿ | ÿ | ÿ | ÿ | ÿ | ÿ | ÿ | ÿ |
| 1. …because there is no specific coding for CIAS. | ÿ | ÿ | ÿ | ÿ | ÿ | ÿ | ÿ | ÿ | ÿ | ÿ |
| 1. ....because CIAS treatments have long waiting lists/are not available. | ÿ | ÿ | ÿ | ÿ | ÿ | ÿ | ÿ | ÿ | ÿ | ÿ |
| 1. …they don’t feel confident in their knowledge to diagnose CIAS. | ÿ | ÿ | ÿ | ÿ | ÿ | ÿ | ÿ | ÿ | ÿ | ÿ |
| 1. …they are not aware that CIAS is a component of schizophrenia. | ÿ | ÿ | ÿ | ÿ | ÿ | ÿ | ÿ | ÿ | ÿ | ÿ |
| 1. …they have difficulty distinguishing CIAS from other symptoms of schizophrenia. | ÿ | ÿ | ÿ | ÿ | ÿ | ÿ | ÿ | ÿ | ÿ | ÿ |
| 1. …they have a lack of guidance about this. | ÿ | ÿ | ÿ | ÿ | ÿ | ÿ | ÿ | ÿ | ÿ | ÿ |
| 1. …they don’t feel confident in their knowledge to explain CIAS well to the patient. | ÿ | ÿ | ÿ | ÿ | ÿ | ÿ | ÿ | ÿ | ÿ | ÿ |
| 1. …they don’t feel confident in their knowledge to treat CIAS. | ÿ | ÿ | ÿ | ÿ | ÿ | ÿ | ÿ | ÿ | ÿ | ÿ |
| 1. …they would prefer to wait until cognitive symptoms have been identified. | ÿ | ÿ | ÿ | ÿ | ÿ | ÿ | ÿ | ÿ | ÿ | ÿ |
| 1. …people with schizophrenia are unlikely to have CIAS. | ÿ | ÿ | ÿ | ÿ | ÿ | ÿ | ÿ | ÿ | ÿ | ÿ |
| 1. …with limited time, they focus their efforts on the treatable aspects of schizophrenia. | ÿ | ÿ | ÿ | ÿ | ÿ | ÿ | ÿ | ÿ | ÿ | ÿ |
| 1. …it is not part of their working protocol to discuss CIAS. | ÿ | ÿ | ÿ | ÿ | ÿ | ÿ | ÿ | ÿ | ÿ | ÿ |
| 1. …they weren’t taught to do this in clinical practice. | ÿ | ÿ | ÿ | ÿ | ÿ | ÿ | ÿ | ÿ | ÿ | ÿ |
| 1. All healthcare practitioners treating with people with schizophrenia inform their patients about cognitive symptoms. | ÿ | ÿ | ÿ | ÿ | ÿ | ÿ | ÿ | ÿ | ÿ | ÿ |

1. **Based on your knowledge and experience, please state your degree of agreement with the following statements related to routinely screening for cognitive symptoms among patients with schizophrenia (i.e. even before noticing a sign or symptom of cognitive impairment in their patient), being 1 completely disagreeing and 9 completely agreeing:** (Please select one answer per row)

| **Screening for cognitive symptoms among patients with schizophrenia** | **1**  **Completely**  **disagree** | **2** | **3** | **4** | **5** | **6** | **7** | **8** | **9**  **Completely**  **agree** | **I do not know / it does not apply to me** |
| --- | --- | --- | --- | --- | --- | --- | --- | --- | --- | --- |
| 1. Some healthcare practitioners treating with people with schizophrenia routinely screen their patients for cognitive symptoms | ÿ | ÿ | ÿ | ÿ | ÿ | ÿ | ÿ | ÿ | ÿ | ÿ |
| There may be different reasons why healthcare practitioners do not routinely screen for cognitive symptoms among their patients with schizophrenia.  In your view, the underlying reason might be... |  |  |  |  |  |  |  |  |  |  |
| 1. …there is no effective treatment for CIAS. | ÿ | ÿ | ÿ | ÿ | ÿ | ÿ | ÿ | ÿ | ÿ | ÿ |
| 1. … there are no established screening protocols for CIAS. | ÿ | ÿ | ÿ | ÿ | ÿ | ÿ | ÿ | ÿ | ÿ | ÿ |
| 1. …there are no standardized screening tools for CIAS. | ÿ | ÿ | ÿ | ÿ | ÿ | ÿ | ÿ | ÿ | ÿ | ÿ |
| 1. …the tools to screen for CIAS are long and not adequate for routine clinical practice. | ÿ | ÿ | ÿ | ÿ | ÿ | ÿ | ÿ | ÿ | ÿ | ÿ |
| 1. …the relevance of CIAS is hard to ascertain without understanding its functional impact. | ÿ | ÿ | ÿ | ÿ | ÿ | ÿ | ÿ | ÿ | ÿ | ÿ |
| 1. …appointment times are too brief and other aspects of schizophrenia care must be prioritised. | ÿ | ÿ | ÿ | ÿ | ÿ | ÿ | ÿ | ÿ | ÿ | ÿ |
| 1. …you don’t feel confident enough in your knowledge about CIAS, to screen for it. | ÿ | ÿ | ÿ | ÿ | ÿ | ÿ | ÿ | ÿ | ÿ | ÿ |
| 1. …you have not received any training regarding how to screen for CIAS. | ÿ | ÿ | ÿ | ÿ | ÿ | ÿ | ÿ | ÿ | ÿ | ÿ |

1. **Based on your knowledge and experience, please state your degree of agreement with the following statements related to patient pathway resources related to CIAS, being 1 completely disagreeing and 9 completely agreeing:** (Please select one answer per row)

| **Resources related to CIAS** | **1**  **Completely**  **disagree** | **2** | **3** | **4** | **5** | **6** | **7** | **8** | **9**  **Completely**  **agree** | **I do not know / it does not apply to me** |
| --- | --- | --- | --- | --- | --- | --- | --- | --- | --- | --- |
| 1. There are no validated criteria to screen for CIAS. | ÿ | ÿ | ÿ | ÿ | ÿ | ÿ | ÿ | ÿ | ÿ | ÿ |
| 1. There are no validated criteria to diagnose CIAS. | ÿ | ÿ | ÿ | ÿ | ÿ | ÿ | ÿ | ÿ | ÿ | ÿ |
| 1. There is a need for validated criteria to screen for CIAS. | ÿ | ÿ | ÿ | ÿ | ÿ | ÿ | ÿ | ÿ | ÿ | ÿ |
| 1. There is a need for validated criteria to diagnose CIAS. | ÿ | ÿ | ÿ | ÿ | ÿ | ÿ | ÿ | ÿ | ÿ | ÿ |
| 1. There are no validated criteria to measure response to CIAS treatments. | ÿ | ÿ | ÿ | ÿ | ÿ | ÿ | ÿ | ÿ | ÿ | ÿ |
| 1. Well-known and well‑validated tools are available, but they are not free to use and therefore not easily accessible to healthcare practitioners. | ÿ | ÿ | ÿ | ÿ | ÿ | ÿ | ÿ | ÿ | ÿ | ÿ |
| 1. Well-known and well‑validated tools are freely available and are being used by healthcare practitioners. | ÿ | ÿ | ÿ | ÿ | ÿ | ÿ | ÿ | ÿ | ÿ | ÿ |
| 1. There is a need for validated criteria to measure response to CIAS treatments. | ÿ | ÿ | ÿ | ÿ | ÿ | ÿ | ÿ | ÿ | ÿ | ÿ |

1. **Based on your knowledge and experience, please state your degree of agreement with the following statements related to the need to improve awareness of CIAS, being 1 completely disagreeing and 9 completely agreeing:** (Please select one answer per row)

| **Needs to improve awareness of CIAS** | **1**  **Completely**  **disagree** | **2** | **3** | **4** | **5** | **6** | **7** | **8** | **9**  **Completely**  **agree** | **I do not know / it does not apply to me** |
| --- | --- | --- | --- | --- | --- | --- | --- | --- | --- | --- |
| 1. There is a need to increase awareness of cognitive impairment associated with schizophrenia among psychiatric healthcare practitioners. | ÿ | ÿ | ÿ | ÿ | ÿ | ÿ | ÿ | ÿ | ÿ | ÿ |
| 1. There is a need to increase awareness of cognitive impairment associated with schizophrenia among non‑psychiatric healthcare practitioners. | ÿ | ÿ | ÿ | ÿ | ÿ | ÿ | ÿ | ÿ | ÿ | ÿ |
| 1. There is a need to increase awareness of CIAS among people with schizophrenia. | ÿ | ÿ | ÿ | ÿ | ÿ | ÿ | ÿ | ÿ | ÿ | ÿ |
| 1. There is a need to increase awareness of CIAS among relatives/caregivers. | ÿ | ÿ | ÿ | ÿ | ÿ | ÿ | ÿ | ÿ | ÿ | ÿ |
| 1. There is a need to increase awareness of CIAS in society. | ÿ | ÿ | ÿ | ÿ | ÿ | ÿ | ÿ | ÿ | ÿ | ÿ |
| 1. There is a need for more continuous medical education programmes about CIAS for healthcare practitioners. | ÿ | ÿ | ÿ | ÿ | ÿ | ÿ | ÿ | ÿ | ÿ | ÿ |

1. **Based on your knowledge and experience, please state your degree of agreement with the following statements related to how to improve CIAS monitoring and follow-up, being 1 completely disagreeing and 9 completely agreeing:** (Please select one answer per row)

| **The following option would help to improve CIAS monitoring and follow-up** | **1**  **Completely**  **disagree** | **2** | **3** | **4** | **5** | **6** | **7** | **8** | **9**  **Completely**  **agree** | **I do not know / it does not apply to me** |
| --- | --- | --- | --- | --- | --- | --- | --- | --- | --- | --- |
| 1. A new short and validated assessment tool | ÿ | ÿ | ÿ | ÿ | ÿ | ÿ | ÿ | ÿ | ÿ | ÿ |
| 1. Continuous education for healthcare practitioners about CIAS | ÿ | ÿ | ÿ | ÿ | ÿ | ÿ | ÿ | ÿ | ÿ | ÿ |
| 1. Education for the general public about CIAS | ÿ | ÿ | ÿ | ÿ | ÿ | ÿ | ÿ | ÿ | ÿ | ÿ |
| 1. Education for people with schizophrenia about cognitive symptoms | ÿ | ÿ | ÿ | ÿ | ÿ | ÿ | ÿ | ÿ | ÿ | ÿ |
| 1. Education for family members about CIAS | ÿ | ÿ | ÿ | ÿ | ÿ | ÿ | ÿ | ÿ | ÿ | ÿ |
| 1. Efforts to increase awareness of CIAS among patient advocacy groups | ÿ | ÿ | ÿ | ÿ | ÿ | ÿ | ÿ | ÿ | ÿ | ÿ |
| 1. New tools and materials (e.g. smartphone apps) for patients with schizophrenia to measure cognitive abilities. | ÿ | ÿ | ÿ | ÿ | ÿ | ÿ | ÿ | ÿ | ÿ | ÿ |
| 1. Availability of a new effective pharmacological treatment for CIAS. | ÿ | ÿ | ÿ | ÿ | ÿ | ÿ | ÿ | ÿ | ÿ | ÿ |

1. **In your regular practice, please indicate which of the following tools you use to screen for, diagnose, or monitor CIAS (if any):** (Please select all that apply)

|  | **Screening** | **Diagnosis** | **Monitoring** | **Not applicable to my job role** |
| --- | --- | --- | --- | --- |
| Assessment of Motor and Processing Skills (AMPS) Assessment | ÿ | ÿ | ÿ | ÿ |
| ACE3 (Addenbrooke's Cognitive Examination) | ÿ | ÿ | ÿ | ÿ |
| BACS (Brief Assessment for Cognition in Schizophrenia) | ÿ | ÿ | ÿ | ÿ |
| d2-test | ÿ | ÿ | ÿ | ÿ |
| Digit Span Memory Test | ÿ | ÿ | ÿ | ÿ |
| GAIN (Global Appraisal of Individual Needs) Cognitive Impairment Scale (CIS) | ÿ | ÿ | ÿ | ÿ |
| MCCB (MATRICS™ Consensus Cognitive Battery) | ÿ | ÿ | ÿ | ÿ |
| Mini-Mental State Examination (MMSE) | ÿ | ÿ | ÿ | ÿ |
| MoCA (Montreal Cognitive Assessment) | ÿ | ÿ | ÿ | ÿ |
| MODA (Memory Orientation and Dementia Assessment) | ÿ | ÿ | ÿ | ÿ |
| Screen for cognitive impairment (SCIP) | ÿ | ÿ | ÿ | ÿ |
| Test of Practical Judgment - Short Form (TPRSF) | ÿ | ÿ | ÿ | ÿ |
| Tower of London Scale | ÿ | ÿ | ÿ | ÿ |
| Other (insert free text):_________________ | ÿ | ÿ | ÿ | ÿ |

1. **Based on your knowledge and experience, please state your degree of agreement with the following statements related to the assessment for CIAS, being 1 completely disagreeing and 9 completely agreeing:** (Please select one answer per row)

| **Thinking about CIAS, a comprehensive assessment of cognition should include the measurement of…** | **1**  **Completely**  **disagree** | **2** | **3** | **4** | **5** | **6** | **7** | **8** | **9**  **Completely**  **agree** | **I do not know / it does not apply to me** |
| --- | --- | --- | --- | --- | --- | --- | --- | --- | --- | --- |
| 1. Attention and concentration | ÿ | ÿ | ÿ | ÿ | ÿ | ÿ | ÿ | ÿ | ÿ | ÿ |
| 1. Working memory | ÿ | ÿ | ÿ | ÿ | ÿ | ÿ | ÿ | ÿ | ÿ | ÿ |
| 1. Verbal memory | ÿ | ÿ | ÿ | ÿ | ÿ | ÿ | ÿ | ÿ | ÿ | ÿ |
| 1. Visual memory | ÿ | ÿ | ÿ | ÿ | ÿ | ÿ | ÿ | ÿ | ÿ | ÿ |
| 1. Processing speed | ÿ | ÿ | ÿ | ÿ | ÿ | ÿ | ÿ | ÿ | ÿ | ÿ |
| 1. Executive functioning: planning, problem-solving, cognitive flexibility and decision-making skills | ÿ | ÿ | ÿ | ÿ | ÿ | ÿ | ÿ | ÿ | ÿ | ÿ |
| 1. Language skills | ÿ | ÿ | ÿ | ÿ | ÿ | ÿ | ÿ | ÿ | ÿ | ÿ |
| 1. Visuospatial abilities | ÿ | ÿ | ÿ | ÿ | ÿ | ÿ | ÿ | ÿ | ÿ | ÿ |
| 1. Motor skills | ÿ | ÿ | ÿ | ÿ | ÿ | ÿ | ÿ | ÿ | ÿ | ÿ |
| 1. Social cognition | ÿ | ÿ | ÿ | ÿ | ÿ | ÿ | ÿ | ÿ | ÿ | ÿ |
| 1. Insight into their condition | ÿ | ÿ | ÿ | ÿ | ÿ | ÿ | ÿ | ÿ | ÿ | ÿ |
| 1. Abstract thinking | ÿ | ÿ | ÿ | ÿ | ÿ | ÿ | ÿ | ÿ | ÿ | ÿ |
| 1. Affect recognition | ÿ | ÿ | ÿ | ÿ | ÿ | ÿ | ÿ | ÿ | ÿ | ÿ |
| 1. Day-to-day functioning: self-care | ÿ | ÿ | ÿ | ÿ | ÿ | ÿ | ÿ | ÿ | ÿ | ÿ |
| 1. Day-to-day functioning: social interactions | ÿ | ÿ | ÿ | ÿ | ÿ | ÿ | ÿ | ÿ | ÿ | ÿ |
| 1. Day-to-day functioning: management of own finances | ÿ | ÿ | ÿ | ÿ | ÿ | ÿ | ÿ | ÿ | ÿ | ÿ |
| 1. Day-to-day functioning: work / school performance | ÿ | ÿ | ÿ | ÿ | ÿ | ÿ | ÿ | ÿ | ÿ | ÿ |

## SECTION 3. Resources Related to CIAS

**We are now focussing on questions related to the resources used by patients with CIAS, addressing their specific needs and identifying any existing gaps or deficiencies.**

1. **Among your patients with schizophrenia, with or without cognitive impairment, and thinking about a typical patient, please indicate how frequently…**

Please, select the best fitting frequency per row (give a single answer per row)

|  | **Patient with schizophrenia WITHOUT COGNITIVE SYMPTOMS** | **Patient with schizophrenia**  **WITH COGNITIVE SYMPTOMS** |
| --- | --- | --- |
| 1. Visit a Psychiatrist | - Once a month - Every 1 to 2 months - Every 3 to 5 months - Every 6 months - Annually - Rarely - Never - I don’t know | - Once a month - Every 1 to 2 months - Every 3 to 5 months - Every 6 months - Annually - Rarely - Never - I don’t know |
| 1. Visit a Psychologist | - Once a month - Every 1 to 2 months - Every 3 to 5 months - Every 6 months - Annually - Rarely - Never - I don’t know | - Once a month - Every 1 to 2 months - Every 3 to 5 months - Every 6 months - Annually - Rarely - Never - I don’t know |
| 1. Visit a General Practitioner | - Once a month - Every 1 to 2 months - Every 3 to 5 months - Every 6 months - Annually - Rarely - Never - I don’t know | - Once a month - Every 1 to 2 months - Every 3 to 5 months - Every 6 months - Annually - Rarely - Never - I don’t know |
| 1. Visit a Nurse | - Once a month - Every 1 to 2 months - Every 3 to 5 months - Every 6 months - Annually - Rarely - Never - I don’t know | - Once a month - Every 1 to 2 months - Every 3 to 5 months - Every 6 months - Annually - Rarely - Never - I don’t know |
| 1. Visit another healthcare or therapy service (e.g. social worker, pharmacist, group therapy) | - Once a month - Every 1 to 2 months - Every 3 to 5 months - Every 6 months - Annually - Rarely - Never - I don’t know | - Once a month - Every 1 to 2 months - Every 3 to 5 months - Every 6 months - Annually - Rarely - Never - I don’t know |
| 1. Requires extra appointments (not scheduled) | - Once a month - Every 1 to 2 months - Every 3 to 5 months - Every 6 months - Annually - Rarely - Never - I don’t know | - Once a month - Every 1 to 2 months - Every 3 to 5 months - Every 6 months - Annually - Rarely - Never - I don’t know |
| 1. Requires emergency visits | - Once a month - Every 1 to 2 months - Every 3 to 5 months - Every 6 months - Annually - Rarely - Never - I don’t know | - Once a month - Every 1 to 2 months - Every 3 to 5 months - Every 6 months - Annually - Rarely - Never - I don’t know |
| 1. Requires admission to hospital or centre | - Once a month - Every 1 to 2 months - Every 3 to 5 months - Every 6 months - Annually - Rarely - Never - I don’t know | - Once a month - Every 1 to 2 months - Every 3 to 5 months - Every 6 months - Annually - Rarely - Never - I don’t know |
| 1. Needs medical leave from work/school | - Once a month - Every 1 to 2 months - Every 3 to 5 months - Every 6 months - Annually - Rarely - Never - I don’t know | - Once a month - Every 1 to 2 months - Every 3 to 5 months - Every 6 months - Annually - Rarely - Never - I don’t know |

1. **Among your patients with schizophrenia, with or without CIAS, thinking about a typical patient, please indicate some figures** *(you don't need to look at any information, just give a number that might fit the reality according to your perception/opinion: we just need to know what the trend is)*

Please, indicate:

|  | **Patient with schizophrenia WITHOUT COGNITIVE SYMPTOMS** | **Patient with schizophrenia WITH COGNITIVE SYMPTOMS** | **I do not know / it does not apply to me** |
| --- | --- | --- | --- |
| 1. Average length of time per routine visit to psychiatrist (minutes) | Range 0-120 minutes | Range 0-120 minutes | ÿ |
| 1. Average time during the visit to psychiatrist required to assess cognition (minutes) | Range 0-120 minutes | Range 0-120 minutes | ÿ |
| 1. Average number of presentations to the emergency department per year | Range 0-99 times per year | Range 0-99 times per year | ÿ |
| 1. Average number of hospitalisations per year | Range 0-99 times per year | Range 0-99 times per year | ÿ |
| 1. Average number of days of hospital inpatient stay | Range 0-365 days | Range 0-365 days | ÿ |
| 1. Average money spent by the public health system on their prescribed medications per year | Range 0-9999999 | Range 0-9999999 | ÿ |
| 1. Average money spent by the public health system on non‑pharmacological treatment per year | Range 0-9999999 | Range 0-9999999 | ÿ |
| 1. Average money spent by the state/public health system on supported living | Range 0-9999999 | Range 0-9999999 | ÿ |
| 1. Average money spent by the state/public health system on social support at home | Range 0-9999999 | Range 0-9999999 | ÿ |
| 1. Average money spent by the state/public health system on medical support at home | Range 0-9999999 | Range 0-9999999 | ÿ |
| 1. Average money spent by the patient on their prescribed medications per year | Range 0-9999999 | Range 0-9999999 | ÿ |
| 1. Average money spent by the patient on non‑pharmacological treatment per year | Range 0-9999999 | Range 0-9999999 | ÿ |

1. **Based on your knowledge and experience, please state your degree of agreement with the following statements related to the resources needed regarding CIAS, being 1 completely disagreeing and 9 completely agreeing:** (Please select one answer per row)

| **Resources for patients with schizophrenia and cognitive symptoms, compared to those with schizophrenia without cognitive symptoms** | **1**  **Completely**  **disagree** | **2** | **3** | **4** | **5** | **6** | **7** | **8** | **9**  **Completely**  **agree** | **I do not know / it does not apply to me** |
| --- | --- | --- | --- | --- | --- | --- | --- | --- | --- | --- |
| 1. Patients with schizophrenia and cognitive symptoms spend more healthcare resources than schizophrenia patients without cognitive symptoms. | ÿ | ÿ | ÿ | ÿ | ÿ | ÿ | ÿ | ÿ | ÿ | ÿ |
| 1. Patients with schizophrenia and cognitive symptoms spend more social resources than schizophrenia patients without cognitive symptoms. | ÿ | ÿ | ÿ | ÿ | ÿ | ÿ | ÿ | ÿ | ÿ | ÿ |
| 1. Patients with schizophrenia and cognitive symptoms need more healthcare resources at their disposal. | ÿ | ÿ | ÿ | ÿ | ÿ | ÿ | ÿ | ÿ | ÿ | ÿ |
| 1. Patients with schizophrenia and cognitive symptoms need more social resources at their disposal. | ÿ | ÿ | ÿ | ÿ | ÿ | ÿ | ÿ | ÿ | ÿ | ÿ |
| 1. Patients with schizophrenia and cognitive symptoms need more formal care resources at their disposal. | ÿ | ÿ | ÿ | ÿ | ÿ | ÿ | ÿ | ÿ | ÿ | ÿ |
| 1. Patients with schizophrenia and cognitive symptoms need more informal care resources at their disposal. | ÿ | ÿ | ÿ | ÿ | ÿ | ÿ | ÿ | ÿ | ÿ | ÿ |
| 1. Families of patients with schizophrenia and cognitive symptoms need more social resources at their disposal. | ÿ | ÿ | ÿ | ÿ | ÿ | ÿ | ÿ | ÿ | ÿ | ÿ |
| 1. The economic impact on society of under-care of cognitive impairment in patients with schizophrenia is high. | ÿ | ÿ | ÿ | ÿ | ÿ | ÿ | ÿ | ÿ | ÿ | ÿ |

## SECTION 4. CIAS Treatment

**This section is focussed on CIAS treatment.**

1. **Based on your knowledge and experience, please state your degree of agreement with the following statements related to the treatment of CIAS, being 1 completely disagreeing and 9 completely agreeing:** (Please select one answer per row)

| **CIAS treatment** | **1**  **Completely**  **disagree** | **2** | **3** | **4** | **5** | **6** | **7** | **8** | **9**  **Completely**  **agree** | **I do not know / it does not apply to me** |
| --- | --- | --- | --- | --- | --- | --- | --- | --- | --- | --- |
| 1. Protocols specifically addressing the treatment of CIAS are needed. | ÿ | ÿ | ÿ | ÿ | ÿ | ÿ | ÿ | ÿ | ÿ | ÿ |
| 1. I already follow a standardised guideline/protocol to treat CIAS. | ÿ | ÿ | ÿ | ÿ | ÿ | ÿ | ÿ | ÿ | ÿ | ÿ |
| 1. There are already effective pharmacological treatments that can be used to treat CIAS. | ÿ | ÿ | ÿ | ÿ | ÿ | ÿ | ÿ | ÿ | ÿ | ÿ |
| 1. There are effective non‑pharmacological treatments for CIAS. | ÿ | ÿ | ÿ | ÿ | ÿ | ÿ | ÿ | ÿ | ÿ | ÿ |
| 1. More research is needed to know which pharmacological treatments for CIAS are effective. | ÿ | ÿ | ÿ | ÿ | ÿ | ÿ | ÿ | ÿ | ÿ | ÿ |
| 1. More research is needed to know which non‑pharmacological treatments for CIAS are effective. | ÿ | ÿ | ÿ | ÿ | ÿ | ÿ | ÿ | ÿ | ÿ | ÿ |
| 1. A new effective pharmacological treatment for CIAS is needed. | ÿ | ÿ | ÿ | ÿ | ÿ | ÿ | ÿ | ÿ | ÿ | ÿ |
| 1. The anticholinergic burden of schizophrenia medications makes CIAS worse. | ÿ | ÿ | ÿ | ÿ | ÿ | ÿ | ÿ | ÿ | ÿ | ÿ |
| 1. Appropriate interventions targeting positive and/or negative symptoms have a positive impact on CIAS. | ÿ | ÿ | ÿ | ÿ | ÿ | ÿ | ÿ | ÿ | ÿ | ÿ |
| 1. Appropriate interventions targeting poor sleep, substance misuse, and/or social isolation have a positive impact on CIAS. | ÿ | ÿ | ÿ | ÿ | ÿ | ÿ | ÿ | ÿ | ÿ | ÿ |

1. **Based on your knowledge and experience, please state your degree of agreement with the following statements related to the pharmacological treatment of CIAS, being 1 completely disagreeing and 9 completely agreeing:** (Please select one answer per row)

| **In your experience, the following pharmacological treatment for CIAS is effective in real-world clinical practice (even if used off‑label)** | **1**  **Completely**  **disagree** | **2** | **3** | **4** | **5** | **6** | **7** | **8** | **9**  **Completely**  **agree** | **I do not know / it does not apply to me** |
| --- | --- | --- | --- | --- | --- | --- | --- | --- | --- | --- |
| 1. Vortioxetine is effective to treat CIAS. | ÿ | ÿ | ÿ | ÿ | ÿ | ÿ | ÿ | ÿ | ÿ | ÿ |
| 1. Methylphenidate is effective to treat CIAS. | ÿ | ÿ | ÿ | ÿ | ÿ | ÿ | ÿ | ÿ | ÿ | ÿ |
| 1. Avoidance of anticholinergics is advisable to not induce cognitive symptoms by secondary drug effects. | ÿ | ÿ | ÿ | ÿ | ÿ | ÿ | ÿ | ÿ | ÿ | ÿ |
| 1. Switching antipsychotics to those with partial agonist properties. | ÿ | ÿ | ÿ | ÿ | ÿ | ÿ | ÿ | ÿ | ÿ | ÿ |
| 1. Other (please specify:_____) | ÿ | ÿ | ÿ | ÿ | ÿ | ÿ | ÿ | ÿ | ÿ | ÿ |

1. **Based on your knowledge and experience, please state your degree of agreement with the following statements related to the prescription of pharmacological treatment for CIAS, being 1 completely disagreeing and 9 completely agreeing:** (Please select one answer per row)

| **In my area, the majority of my colleagues consider the following pharmacological treatments for CIAS patients…** | **1**  **Completely**  **disagree** | **2** | **3** | **4** | **5** | **6** | **7** | **8** | **9**  **Completely**  **agree** | **I do not know / it does not apply to me** |
| --- | --- | --- | --- | --- | --- | --- | --- | --- | --- | --- |
| 1. Vortioxetine | ÿ | ÿ | ÿ | ÿ | ÿ | ÿ | ÿ | ÿ | ÿ | ÿ |
| 1. Methylphenidate | ÿ | ÿ | ÿ | ÿ | ÿ | ÿ | ÿ | ÿ | ÿ | ÿ |
| 1. Avoidance of anticholinergics | ÿ | ÿ | ÿ | ÿ | ÿ | ÿ | ÿ | ÿ | ÿ | ÿ |
| 1. Switching antipsychotics to those with partial agonist properties | ÿ | ÿ | ÿ | ÿ | ÿ | ÿ | ÿ | ÿ | ÿ | ÿ |
| 1. Other (please specify:_____) | ÿ | ÿ | ÿ | ÿ | ÿ | ÿ | ÿ | ÿ | ÿ | ÿ |

1. **Based on your knowledge and experience, please let us know the effectiveness of non-pharmacological treatment of CIAS:** (Please select one answer per row)

| **The following non‑pharmacological treatment for CIAS is effective in real-world clinical practice …** | **Non effective at all** | **Ineffective** | **Neutral** | **Effective** | **Very Effective** | **I do not know / it does not apply to me** |
| --- | --- | --- | --- | --- | --- | --- |
| 1. Cognitive Remediation Therapy (CRT) | ÿ | ÿ | ÿ | ÿ | ÿ | ÿ |
| 1. Cognitive Behavioural Therapy (CBT) | ÿ | ÿ | ÿ | ÿ | ÿ | ÿ |
| 1. Eye movement desensitization reprogramming (EMDR) | ÿ | ÿ | ÿ | ÿ | ÿ | ÿ |
| 1. Transcranial Magnetic Stimulation (TMS) / Transcranial Direct Current Stimulation (tDCS) | ÿ | ÿ | ÿ | ÿ | ÿ | ÿ |
| 1. Psychoeducation | ÿ | ÿ | ÿ | ÿ | ÿ | ÿ |
| 1. Psychoanalysis | ÿ | ÿ | ÿ | ÿ | ÿ | ÿ |
| 1. Motivational interviewing | ÿ | ÿ | ÿ | ÿ | ÿ | ÿ |
| 1. Social Rehabilitation / Social Skills Training (e.g. encouraging voluntary work, social groups) | ÿ | ÿ | ÿ | ÿ | ÿ | ÿ |
| 1. Occupational Interventions | ÿ | ÿ | ÿ | ÿ | ÿ | ÿ |
| 1. Addressing Substance Use | ÿ | ÿ | ÿ | ÿ | ÿ | ÿ |
| 1. Addressing Sleep | ÿ | ÿ | ÿ | ÿ | ÿ | ÿ |
| 1. Addressing Metabolic Health / Physical Exercise | ÿ | ÿ | ÿ | ÿ | ÿ | ÿ |

1. **Based on your knowledge and experience, please let us know the accessibility of the following non‑pharmacological treatments for CIAS in your area:** (Please select one answer per row)

| **In my area, the following non‑pharmacological treatment is accessible for CIAS patients who need it…** | **Not available at all** | **Limited availability due to limited capacity of therapists** | **Only available privately** | **Available with partial public reimbursement** | **Available free of charge** | **I do not know / it does not apply to me** |
| --- | --- | --- | --- | --- | --- | --- |
| 1. Cognitive Remediation Therapy (CRT) | ÿ | ÿ | ÿ | ÿ | ÿ | ÿ |
| 1. Cognitive Behavioural Therapy (CBT) | ÿ | ÿ | ÿ | ÿ | ÿ | ÿ |
| 1. Eye movement desensitization reprogramming (EMDR) | ÿ | ÿ | ÿ | ÿ | ÿ | ÿ |
| 1. Transcranial Magnetic Stimulation (TMS) / Transcranial Direct Current Stimulation (tDCS) | ÿ | ÿ | ÿ | ÿ | ÿ | ÿ |
| 1. Psychoeducation | ÿ | ÿ | ÿ | ÿ | ÿ | ÿ |
| 1. Psychoanalysis | ÿ | ÿ | ÿ | ÿ | ÿ | ÿ |
| 1. Motivational interviewing | ÿ | ÿ | ÿ | ÿ | ÿ | ÿ |
| 1. Social Rehabilitation / Social Skills Training (e.g. encouraging voluntary work, social groups) | ÿ | ÿ | ÿ | ÿ | ÿ | ÿ |
| 1. Occupational Interventions | ÿ | ÿ | ÿ | ÿ | ÿ | ÿ |
| 1. Addressing Substance Use | ÿ | ÿ | ÿ | ÿ | ÿ | ÿ |
| 1. Addressing Sleep | ÿ | ÿ | ÿ | ÿ | ÿ | ÿ |
| 1. Addressing Metabolic Health / Physical Exercise | ÿ | ÿ | ÿ | ÿ | ÿ | ÿ |

1. **Based on your knowledge and experience, please state your degree of agreement with the following statements related to the prescription of non-pharmacological treatment for CIAS, being 1 completely disagreeing and 9 completely agreeing:** (Please select one answer per row)

| **In my area, the majority of my colleagues consider prescribing the following non‑pharmacological treatments for CIAS patients who need it…** | **1**  **Completely**  **disagree** | **2** | **3** | **4** | **5** | **6** | **7** | **8** | **9**  **Completely**  **agree** | **I do not know / it does not apply to me** |
| --- | --- | --- | --- | --- | --- | --- | --- | --- | --- | --- |
| 1. Cognitive Remediation Therapy (CRT) | ÿ | ÿ | ÿ | ÿ | ÿ | ÿ | ÿ | ÿ | ÿ | ÿ |
| 1. Cognitive Behavioural Therapy (CBT) | ÿ | ÿ | ÿ | ÿ | ÿ | ÿ | ÿ | ÿ | ÿ | ÿ |
| 1. Eye movement desensitization reprogramming (EMDR) | ÿ | ÿ | ÿ | ÿ | ÿ | ÿ | ÿ | ÿ | ÿ | ÿ |
| 1. Transcranial Magnetic Stimulation (TMS) / Transcranial Direct Current Stimulation (tDCS) | ÿ | ÿ | ÿ | ÿ | ÿ | ÿ | ÿ | ÿ | ÿ | ÿ |
| 1. Psychoeducation | ÿ | ÿ | ÿ | ÿ | ÿ | ÿ | ÿ | ÿ | ÿ | ÿ |
| 1. Psychoanalysis | ÿ | ÿ | ÿ | ÿ | ÿ | ÿ | ÿ | ÿ | ÿ | ÿ |
| 1. Motivational interviewing | ÿ | ÿ | ÿ | ÿ | ÿ | ÿ | ÿ | ÿ | ÿ | ÿ |
| 1. Social Rehabilitation / Social Skills Training (e.g. encouraging voluntary work, social groups) | ÿ | ÿ | ÿ | ÿ | ÿ | ÿ | ÿ | ÿ | ÿ | ÿ |
| 1. Occupational Interventions | ÿ | ÿ | ÿ | ÿ | ÿ | ÿ | ÿ | ÿ | ÿ | ÿ |
| 1. Addressing Substance Use | ÿ | ÿ | ÿ | ÿ | ÿ | ÿ | ÿ | ÿ | ÿ | ÿ |
| 1. Addressing Sleep | ÿ | ÿ | ÿ | ÿ | ÿ | ÿ | ÿ | ÿ | ÿ | ÿ |
| 1. Addressing Metabolic Health / Physical Exercise | ÿ | ÿ | ÿ | ÿ | ÿ | ÿ | ÿ | ÿ | ÿ | ÿ |

1. **Based on your knowledge and experience, please state your degree of agreement with the following statements related to the future treatment for CIAS, being 1 completely disagreeing and 9 completely agreeing:** (Please select one answer per row)

| **If an effective pharmacological treatment for CIAS becomes available in the future…** | **1**  **Completely**  **disagree** | **2** | **3** | **4** | **5** | **6** | **7** | **8** | **9**  **Completely**  **agree** | **I do not know / it does not apply to me** |
| --- | --- | --- | --- | --- | --- | --- | --- | --- | --- | --- |
| 1. Non-pharmacological treatments will still need to be prescribed alongside it. | ÿ | ÿ | ÿ | ÿ | ÿ | ÿ | ÿ | ÿ | ÿ | ÿ |
| 1. It should be prescribed by Psychiatrists. | ÿ | ÿ | ÿ | ÿ | ÿ | ÿ | ÿ | ÿ | ÿ | ÿ |
| 1. General Practitioners (GPs) should be allowed to renew its prescription. | ÿ | ÿ | ÿ | ÿ | ÿ | ÿ | ÿ | ÿ | ÿ | ÿ |
| 1. It should be fully reimbursed by the national health system. | ÿ | ÿ | ÿ | ÿ | ÿ | ÿ | ÿ | ÿ | ÿ | ÿ |
| 1. The care pathway of schizophrenia should be optimised to include the analysis, treatment and monitoring of CIAS. | ÿ | ÿ | ÿ | ÿ | ÿ | ÿ | ÿ | ÿ | ÿ | ÿ |

1. **Based on your knowledge and experience, please state your degree of agreement with the following statements related to the future treatment for CIAS, being 1 completely disagreeing and 9 completely agreeing:** (Please select one answer per row)

| **Improvements that a new pharmacological treatment could provide** | **1**  **Completely**  **disagree** | **2** | **3** | **4** | **5** | **6** | **7** | **8** | **9**  **Completely**  **agree** | **I do not know / it does not apply to me** |
| --- | --- | --- | --- | --- | --- | --- | --- | --- | --- | --- |
| 1. An acceptable treatment effect would be Cohen’s d of 0.3 vs placebo (similar to the effect seen with SSRIs to treat depression). | ÿ | ÿ | ÿ | ÿ | ÿ | ÿ | ÿ | ÿ | ÿ | ÿ |
| 1. An acceptable treatment effect would be Cohen’s d of 0.6 vs placebo (similar to the effect seen with SSRIs to treat depression). | ÿ | ÿ | ÿ | ÿ | ÿ | ÿ | ÿ | ÿ | ÿ | ÿ |
| 1. An acceptable treatment effect would be Cohen’s d of greater than 0.6 vs placebo (similar to the effect seen with SSRIs to treat depression). | ÿ | ÿ | ÿ | ÿ | ÿ | ÿ | ÿ | ÿ | ÿ | ÿ |
| 1. The introduction of a new depot treatment for CIAS could improve adherence. | ÿ | ÿ | ÿ | ÿ | ÿ | ÿ | ÿ | ÿ | ÿ | ÿ |
| 1. The introduction of an oral treatment with a long half-life for CIAS could improve adherence. | ÿ | ÿ | ÿ | ÿ | ÿ | ÿ | ÿ | ÿ | ÿ | ÿ |

1. **If an effective treatment for CIAS becomes available in the future, how much time in each patient consultation could you spend to assess cognition?**

*_____________minutes per patient consultation* **(Range 0-120 minutes).**

SECTION 5. CIAS Burden

**We are now focussing on questions related to burdens associated with CIAS.**

1. **Based on your knowledge and experience, please state your degree of agreement with the following statements related to the health implications of CIAS, being 1 completely disagreeing and 9 completely agreeing:** (Please select one answer per row)

| **As compared to people with schizophrenia and not cognitive symptoms, people with schizophrenia who DO have cognitive symptoms experience…** | **1**  **Completely**  **disagree** | **2** | **3** | **4** | **5** | **6** | **7** | **8** | **9**  **Completely**  **agree** | **I do not know / it does not apply to me** |
| --- | --- | --- | --- | --- | --- | --- | --- | --- | --- | --- |
| 1. Higher incidence of other mental health conditions. | ÿ | ÿ | ÿ | ÿ | ÿ | ÿ | ÿ | ÿ | ÿ | ÿ |
| 1. Higher incidence of physical disease/comorbidity (e.g. cardiometabolic syndrome, etc). | ÿ | ÿ | ÿ | ÿ | ÿ | ÿ | ÿ | ÿ | ÿ | ÿ |
| 1. Higher rates of psychotic episodes. | ÿ | ÿ | ÿ | ÿ | ÿ | ÿ | ÿ | ÿ | ÿ | ÿ |
| 1. Higher rates of attempting suicide. | ÿ | ÿ | ÿ | ÿ | ÿ | ÿ | ÿ | ÿ | ÿ | ÿ |
| 1. Higher rates of completing suicide. | ÿ | ÿ | ÿ | ÿ | ÿ | ÿ | ÿ | ÿ | ÿ | ÿ |
| 1. Shortened length of life. | ÿ | ÿ | ÿ | ÿ | ÿ | ÿ | ÿ | ÿ | ÿ | ÿ |
| 1. Shortened length of physically healthy life. | ÿ | ÿ | ÿ | ÿ | ÿ | ÿ | ÿ | ÿ | ÿ | ÿ |

1. **Based on your knowledge and experience, please state your degree of agreement with the following statements related to specific comorbidities associated with CIAS, being 1 completely disagreeing and 9 completely agreeing:** (Please select one answer per row)

| **As compared to schizophrenia without cognitive symptoms, CIAS is associated with an increased risk of…** | **1**  **Completely**  **disagree** | **2** | **3** | **4** | **5** | **6** | **7** | **8** | **9**  **Completely**  **agree** | **I do not know / it does not apply to me** |
| --- | --- | --- | --- | --- | --- | --- | --- | --- | --- | --- |
| 1. Cancers | ÿ | ÿ | ÿ | ÿ | ÿ | ÿ | ÿ | ÿ | ÿ | ÿ |
| 1. Type II Diabetes | ÿ | ÿ | ÿ | ÿ | ÿ | ÿ | ÿ | ÿ | ÿ | ÿ |
| 1. Obesity | ÿ | ÿ | ÿ | ÿ | ÿ | ÿ | ÿ | ÿ | ÿ | ÿ |
| 1. Hypertension | ÿ | ÿ | ÿ | ÿ | ÿ | ÿ | ÿ | ÿ | ÿ | ÿ |
| 1. Cardiovascular comorbidities | ÿ | ÿ | ÿ | ÿ | ÿ | ÿ | ÿ | ÿ | ÿ | ÿ |
| 1. Metabolic syndrome | ÿ | ÿ | ÿ | ÿ | ÿ | ÿ | ÿ | ÿ | ÿ | ÿ |
| 1. Increased infection frequency / severity | ÿ | ÿ | ÿ | ÿ | ÿ | ÿ | ÿ | ÿ | ÿ | ÿ |
| 1. Sleep disorders | ÿ | ÿ | ÿ | ÿ | ÿ | ÿ | ÿ | ÿ | ÿ | ÿ |
| 1. Alcohol misuse | ÿ | ÿ | ÿ | ÿ | ÿ | ÿ | ÿ | ÿ | ÿ | ÿ |
| 1. Drug misuse | ÿ | ÿ | ÿ | ÿ | ÿ | ÿ | ÿ | ÿ | ÿ | ÿ |
| 1. Anxiety disorders | ÿ | ÿ | ÿ | ÿ | ÿ | ÿ | ÿ | ÿ | ÿ | ÿ |
| 1. Depression | ÿ | ÿ | ÿ | ÿ | ÿ | ÿ | ÿ | ÿ | ÿ | ÿ |
| 1. Other (please specify:______) | ÿ | ÿ | ÿ | ÿ | ÿ | ÿ | ÿ | ÿ | ÿ | ÿ |

1. **Based on your knowledge and experience, please state your degree of agreement with the following statements related to the physical health implications of CIAS, being 1 completely disagreeing and 9 completely agreeing:** (Please select one answer per row)

| **Cognitive symptoms lead people with schizophrenia to experience poorer physical health, because the cognitive symptoms mean they are…** | **1**  **Completely**  **disagree** | **2** | **3** | **4** | **5** | **6** | **7** | **8** | **9**  **Completely**  **agree** | **I do not know / it does not apply to me** |
| --- | --- | --- | --- | --- | --- | --- | --- | --- | --- | --- |
| 1. More likely to have poor personal hygiene. | ÿ | ÿ | ÿ | ÿ | ÿ | ÿ | ÿ | ÿ | ÿ | ÿ |
| 1. More likely to miss early warning signs of physical disease. | ÿ | ÿ | ÿ | ÿ | ÿ | ÿ | ÿ | ÿ | ÿ | ÿ |
| 1. More likely to ascribe legitimate physical health symptoms to psychosis or other factors. | ÿ | ÿ | ÿ | ÿ | ÿ | ÿ | ÿ | ÿ | ÿ | ÿ |
| 1. More likely to forget their physical symptoms long enough to tell the doctor. | ÿ | ÿ | ÿ | ÿ | ÿ | ÿ | ÿ | ÿ | ÿ | ÿ |
| 1. More likely to forget to take their medications. | ÿ | ÿ | ÿ | ÿ | ÿ | ÿ | ÿ | ÿ | ÿ | ÿ |
| 1. Less likely to maintain a healthy diet. | ÿ | ÿ | ÿ | ÿ | ÿ | ÿ | ÿ | ÿ | ÿ | ÿ |
| 1. Less likely to take healthy levels of physical exercise. | ÿ | ÿ | ÿ | ÿ | ÿ | ÿ | ÿ | ÿ | ÿ | ÿ |
| 1. Less likely to explain their physical symptoms clearly to their doctor. | ÿ | ÿ | ÿ | ÿ | ÿ | ÿ | ÿ | ÿ | ÿ | ÿ |
| 1. Less likely to succeed to make and/or attend an appointment with their doctor. | ÿ | ÿ | ÿ | ÿ | ÿ | ÿ | ÿ | ÿ | ÿ | ÿ |
| 1. Less likely to attend appointments with the psychiatrist, GP, dentist and opticians. | ÿ | ÿ | ÿ | ÿ | ÿ | ÿ | ÿ | ÿ | ÿ | ÿ |
| 1. Less likely to have good adherence to schizophrenia treatment. | ÿ | ÿ | ÿ | ÿ | ÿ | ÿ | ÿ | ÿ | ÿ | ÿ |
| 1. Less likely to continue learning about their health. | ÿ | ÿ | ÿ | ÿ | ÿ | ÿ | ÿ | ÿ | ÿ | ÿ |
| 1. Less likely to wear appropriate clothing for the weather. | ÿ | ÿ | ÿ | ÿ | ÿ | ÿ | ÿ | ÿ | ÿ | ÿ |
| 1. Other (please specify:_____) | ÿ | ÿ | ÿ | ÿ | ÿ | ÿ | ÿ | ÿ | ÿ | ÿ |

1. **If you consider that cognitive symptoms are associated with higher rates of psychotic episodes, why do you think this is?** (Please only select one option)

- Because having more psychotic episodes makes CIAS worse
- Because CIAS increases the chance of psychosis recurrence
- Both of the above
- Because CIAS is associated with poor adherence with antipsychotics
- Other (Please specify:________)
- There is no association between CIAS and higher rates of psychotic episodes

1. **Based on your knowledge and experience, please state your degree of agreement with the following statements related to the social implications of CIAS, being 1 completely disagreeing and 9 completely agreeing:** (Please select one answer per row)

| **As compared with people with schizophrenia without cognitive symptoms, people with CIAS…** | **1**  **Completely**  **disagree** | **2** | **3** | **4** | **5** | **6** | **7** | **8** | **9**  **Completely**  **agree** | **I do not know / it does not apply to me** |
| --- | --- | --- | --- | --- | --- | --- | --- | --- | --- | --- |
| 1. Are more socially isolated. | ÿ | ÿ | ÿ | ÿ | ÿ | ÿ | ÿ | ÿ | ÿ | ÿ |
| 1. Avoid social situations out of fear they will forget someone’s name or face. | ÿ | ÿ | ÿ | ÿ | ÿ | ÿ | ÿ | ÿ | ÿ | ÿ |
| 1. Struggle to make new friends. | ÿ | ÿ | ÿ | ÿ | ÿ | ÿ | ÿ | ÿ | ÿ | ÿ |
| 1. Struggle to maintain previous friendships. | ÿ | ÿ | ÿ | ÿ | ÿ | ÿ | ÿ | ÿ | ÿ | ÿ |
| 1. Have conflicts with family members who misinterpret their mistakes as laziness. | ÿ | ÿ | ÿ | ÿ | ÿ | ÿ | ÿ | ÿ | ÿ | ÿ |
| 1. Have conflicts with family members who misinterpret their mistakes as oppositionality. | ÿ | ÿ | ÿ | ÿ | ÿ | ÿ | ÿ | ÿ | ÿ | ÿ |
| 1. Are less likely to date and marry. | ÿ | ÿ | ÿ | ÿ | ÿ | ÿ | ÿ | ÿ | ÿ | ÿ |

1. **Based on your knowledge and experience, please state your degree of agreement with the following statements related to the education and employment implications of CIAS, being 1 completely disagreeing and 9 completely agreeing:** (Please select one answer per row)

| **As compared with people with schizophrenia without cognitive symptoms, people with CIAS…** | **1**  **Completely**  **disagree** | **2** | **3** | **4** | **5** | **6** | **7** | **8** | **9**  **Completely**  **agree** | **I do not know / it does not apply to me** |
| --- | --- | --- | --- | --- | --- | --- | --- | --- | --- | --- |
| 1. Are less likely to do well at school. | ÿ | ÿ | ÿ | ÿ | ÿ | ÿ | ÿ | ÿ | ÿ | ÿ |
| 1. Are less likely to graduate from university. | ÿ | ÿ | ÿ | ÿ | ÿ | ÿ | ÿ | ÿ | ÿ | ÿ |
| 1. Are less likely to obtain a driving license. | ÿ | ÿ | ÿ | ÿ | ÿ | ÿ | ÿ | ÿ | ÿ | ÿ |
| 1. Are less likely to get a new job. | ÿ | ÿ | ÿ | ÿ | ÿ | ÿ | ÿ | ÿ | ÿ | ÿ |
| 1. Are more likely to lose their job. | ÿ | ÿ | ÿ | ÿ | ÿ | ÿ | ÿ | ÿ | ÿ | ÿ |
| 1. Are less likely to get promotions in their job. | ÿ | ÿ | ÿ | ÿ | ÿ | ÿ | ÿ | ÿ | ÿ | ÿ |
| 1. If a worker with CIAS tells their employer of their diagnosis, they are more likely to keep their job. | ÿ | ÿ | ÿ | ÿ | ÿ | ÿ | ÿ | ÿ | ÿ | ÿ |
| 1. Other (please specify:_____) | ÿ | ÿ | ÿ | ÿ | ÿ | ÿ | ÿ | ÿ | ÿ | ÿ |

1. **Based on your knowledge and experience, please state your degree of agreement with the following statements related to the employment implications of CIAS, being 1 completely disagreeing and 9 completely agreeing:** (Please select one answer per row)

| **As compared with people with schizophrenia without cognitive symptoms, people with CIAS are less likely to get a new job or maintain their current job because they…** | **1**  **Completely**  **disagree** | **2** | **3** | **4** | **5** | **6** | **7** | **8** | **9**  **Completely**  **agree** | **I do not know / it does not apply to me** |
| --- | --- | --- | --- | --- | --- | --- | --- | --- | --- | --- |
| 1. Tend to have poorer memory. | ÿ | ÿ | ÿ | ÿ | ÿ | ÿ | ÿ | ÿ | ÿ | ÿ |
| 1. Tend to have difficulty reading and processing information. | ÿ | ÿ | ÿ | ÿ | ÿ | ÿ | ÿ | ÿ | ÿ | ÿ |
| 1. Tend to struggle concentrating longer than a few minutes, e.g. to update a curriculum vitae / succeed in workplace tasks. | ÿ | ÿ | ÿ | ÿ | ÿ | ÿ | ÿ | ÿ | ÿ | ÿ |
| 1. Tend to have difficulty passing the interview due to slow thinking. | ÿ | ÿ | ÿ | ÿ | ÿ | ÿ | ÿ | ÿ | ÿ | ÿ |
| 1. Tend to be slower to notice career opportunities. | ÿ | ÿ | ÿ | ÿ | ÿ | ÿ | ÿ | ÿ | ÿ | ÿ |
| 1. Tend to be stigmatised by their employers. | ÿ | ÿ | ÿ | ÿ | ÿ | ÿ | ÿ | ÿ | ÿ | ÿ |
| 1. Tend to have difficulties with time-keeping and scheduling. | ÿ | ÿ | ÿ | ÿ | ÿ | ÿ | ÿ | ÿ | ÿ | ÿ |

1. **Based on your knowledge and experience, please state your degree of agreement with the following statements related to the financial implications of CIAS, being 1 completely disagreeing and 9 completely agreeing:** (Please select one answer per row)

| **As compared with people with schizophrenia without cognitive symptoms, people with CIAS…** | **1**  **Completely**  **disagree** | **2** | **3** | **4** | **5** | **6** | **7** | **8** | **9**  **Completely**  **agree** | **I do not know / it does not apply to me** |
| --- | --- | --- | --- | --- | --- | --- | --- | --- | --- | --- |
| 1. Are more likely to be in debt. | ÿ | ÿ | ÿ | ÿ | ÿ | ÿ | ÿ | ÿ | ÿ | ÿ |
| 1. Are more likely to be homeless. | ÿ | ÿ | ÿ | ÿ | ÿ | ÿ | ÿ | ÿ | ÿ | ÿ |
| 1. Are more likely to be involved with the legal or judicial system. | ÿ | ÿ | ÿ | ÿ | ÿ | ÿ | ÿ | ÿ | ÿ | ÿ |
| 1. Are more likely to be in receipt of state benefits. | ÿ | ÿ | ÿ | ÿ | ÿ | ÿ | ÿ | ÿ | ÿ | ÿ |
| 1. Are more likely to depend on their family financially. | ÿ | ÿ | ÿ | ÿ | ÿ | ÿ | ÿ | ÿ | ÿ | ÿ |
| 1. Are more likely to require 24-hour supported accommodation. | ÿ | ÿ | ÿ | ÿ | ÿ | ÿ | ÿ | ÿ | ÿ | ÿ |
| 1. Are more likely to require professional caregiver support into old age. | ÿ | ÿ | ÿ | ÿ | ÿ | ÿ | ÿ | ÿ | ÿ | ÿ |
| 1. Cost more to the state financially, in terms of cost of mental health treatments. | ÿ | ÿ | ÿ | ÿ | ÿ | ÿ | ÿ | ÿ | ÿ | ÿ |
| 1. Cost more to the state financially, in terms of physical health costs. | ÿ | ÿ | ÿ | ÿ | ÿ | ÿ | ÿ | ÿ | ÿ | ÿ |
| 1. Cost more to the state financially, in terms of social costs. | ÿ | ÿ | ÿ | ÿ | ÿ | ÿ | ÿ | ÿ | ÿ | ÿ |

1. **Based on your knowledge and experience, please state your degree of agreement with the following statements related to the societal implications of CIAS, being 1 completely disagreeing and 9 completely agreeing:** (Please select one answer per row)

| **Society loses out from the possible benefits that someone with CIAS can bring, in the following ways…** | **1**  **Completely**  **disagree** | **2** | **3** | **4** | **5** | **6** | **7** | **8** | **9**  **Completely**  **agree** | **I do not know / it does not apply to me** |
| --- | --- | --- | --- | --- | --- | --- | --- | --- | --- | --- |
| 1. The experience and skillset they might bring to society | ÿ | ÿ | ÿ | ÿ | ÿ | ÿ | ÿ | ÿ | ÿ | ÿ |
| 1. Payment of income taxes | ÿ | ÿ | ÿ | ÿ | ÿ | ÿ | ÿ | ÿ | ÿ | ÿ |
| 1. Financial and experiential contribution that family caregivers might bring to society | ÿ | ÿ | ÿ | ÿ | ÿ | ÿ | ÿ | ÿ | ÿ | ÿ |
| 1. Loss of their role within the family unit | ÿ | ÿ | ÿ | ÿ | ÿ | ÿ | ÿ | ÿ | ÿ | ÿ |

Thank you very much for participating in this survey. We would like to re-contact some respondents based on your answers in this survey for a simplified survey (wave 2). Would you agree for us to re-contact you in the future about this and similar interviews?

1. Yes, I agree.
2. No, I do not agree. [PROGRAMMING: If selected, please show the following disclaimer*: It is vital for this study to have your participation in the second wave in order to have all the necessary information. Please allow us to contact you again.*]
