# Supplementary material for: Understanding the burden of cognitive impairment associated with schizophrenia: Results from the international LUCIA study
Source: Eur Psychiatry. 2026 Apr 28;69(1):e55. doi: 10.1192/j.eurpsy.2026.12208 (PMC13227134; doi:10.1192/j.eurpsy.2026.12208)
Supplement: Correll et al. supplementary material [file S0924933826122081sup001.zip › SUPPL_MAT_2_Patient_caregiver_Questionnaire.docx]

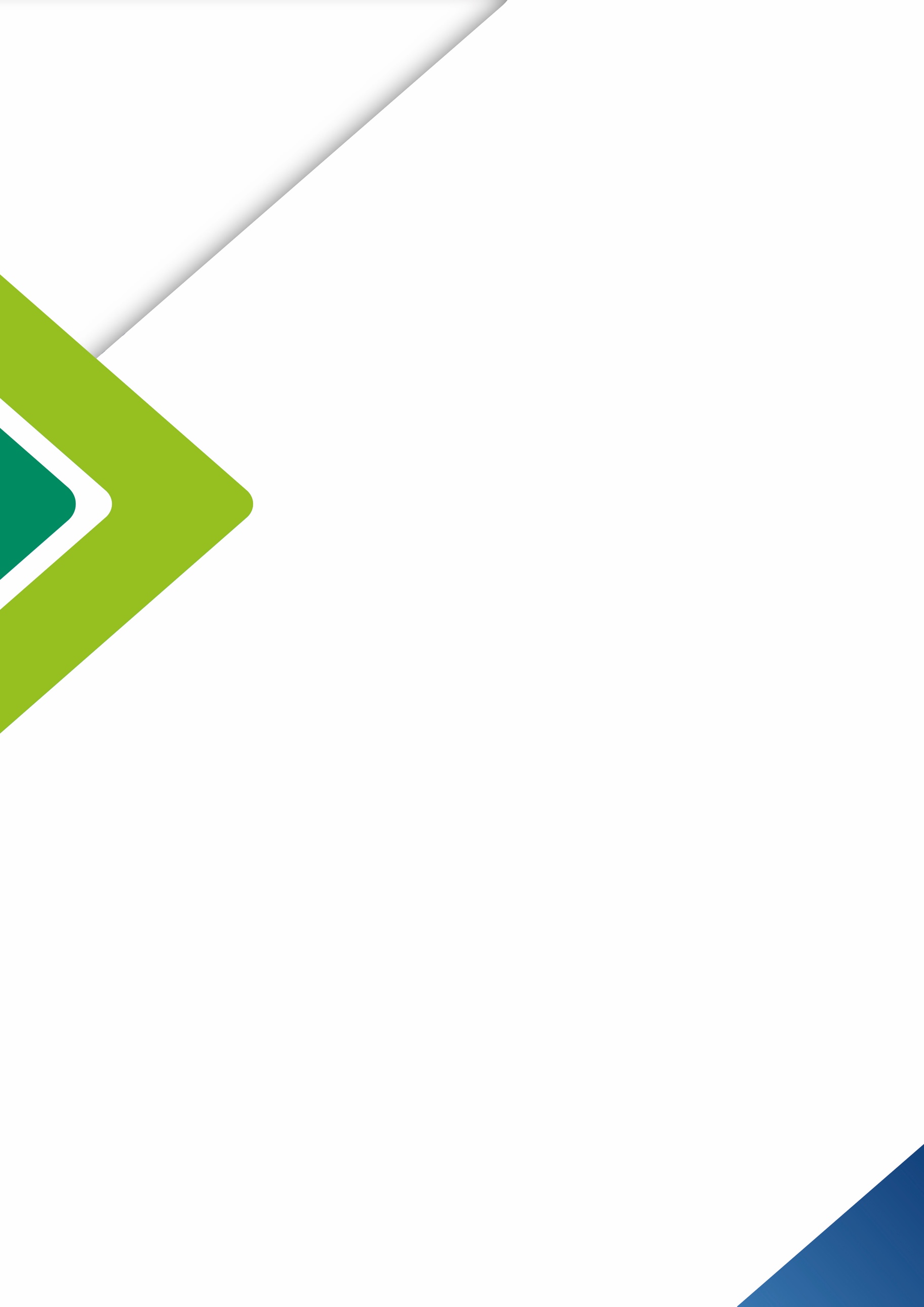


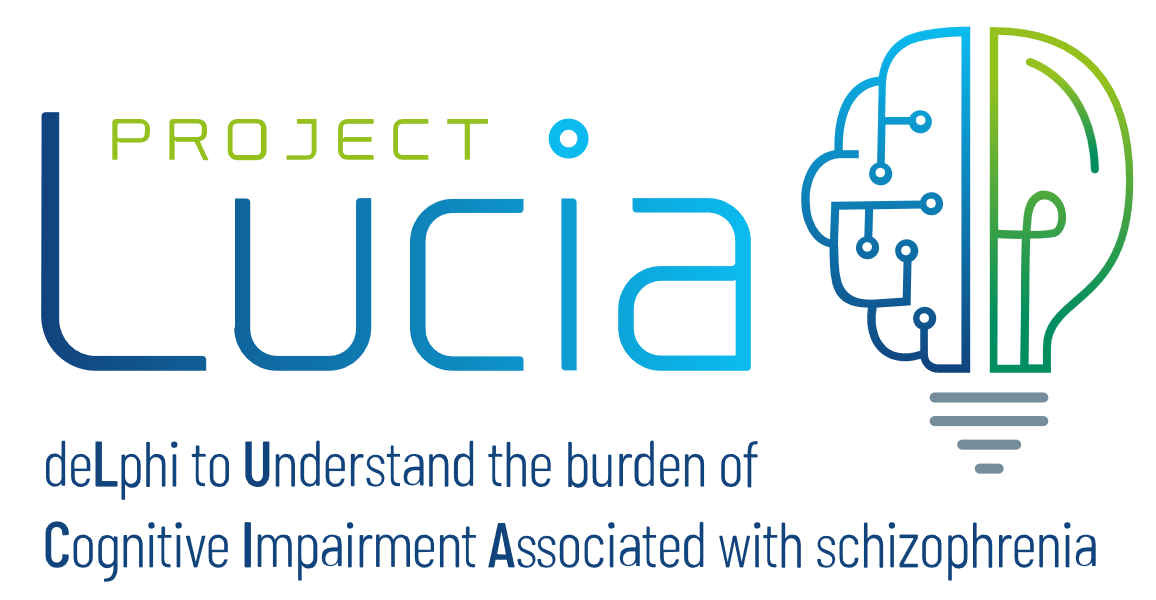


Delphi: Questionnaire for People with CIAS and Caregivers - Final Version

MAY 2024

# Patient and Caregiver Questionnaire (30 mins)

## INTRODUCTION

We are inviting you to take part in a project being run by Adelphi Targis, a research company, and sponsored by a pharmaceutical company. **Before you decide if you want to join the project, please read the following information.**

We are doing a survey research study to gain an **understanding on the impact of certain symptoms in the day‑to‑day life of people living with schizophrenia. This will ultimately help us to identify unmet needs and solutions.** We are conducting this research with people in several countries and this survey will last approximately 30 minutes.

We would like to assure you that we act in accordance with all relevant laws and regulations (the ESOMAR and EphMRA codes of conduct regarding anonymity and confidentiality). **There are no right or wrong answers.** The aim of this study is to understand your experience and opinion.

Any information you tell us will be treated with **the strictest confidentiality and the results are anonymised, so your name will not be asked anywhere.**

We will not be able to identify you from your answers, and all answers from each participant will be grouped together before they are analysed. Data will be accessible by the project team at Adelphi Targis and, according to the law, they will be kept on a secure server for 10 years and then deleted. All data will be handled in accordance with the 2016 General Data Protection Regulation (GDPR). Information on Adelphi’s data protection and GDPR compliance can be found at the following webpages (in English only): [www.adelphitargis.com/en](http://www.adelphitargis.com/en).

- If you would like more information or have questions or concerns about the project, please contact [jasmin.walravens@adelphitargis.com](mailto:jasmin.walravens@adelphitargis.com).
- If you are unhappy with the way your personal data has been handled, you have a right to complain to the Adelphi Targis’ Data Protection Officer at [dpo@adelphitargis.com](mailto:dpo@adelphitargis.com).

Taking part in this study is entirely optional and you can pull out of the survey at any time, without giving a reason, by exiting the browser window. If you exit the survey before it is finished, we may use the answers you have given until that point. If you are willing to take part, then please continue with the survey.

***Results***

Aggregated results from this study will be put together into a report that we will share with the pharmaceutical company. They may also be presented at international conferences and published in medical journals.

***Adverse events***

Although all information that you give us in this survey will be treated confidentially, if you mention any side effect of a medication or any other safety information, e.g., about how safely a medicine is being used, and/or a medicine quality complaint during the completion of this questionnaire, we will need to report it to the company, even if you have already done so.

In such a situation, you will be asked whether you are willing to waive the confidentiality given to you under the Market Research Codes of Conduct specifically in relation to that side effect or other safety information and/or medicine quality complaint. This will allow us to report and look into the problem further. You always have the right to remain anonymous and if you choose not to provide your contact details, you will only be identified by your role (‘patient’ or ‘caregiver’). Any other information you give during the survey will remain confidential.

YOUR CONSENT TO TAKE PART

Please indicate that you have read the information above:

- **Yes,** I have read and understood the above information and I give my consent to take part in this survey. I understand that I can withdraw at any time and without giving a reason.
- **No**, I do not wish to continue the survey *[PROGRAMMING: If selected, please show the following warning message: Please read the information provided and select the ‘Yes’ option in order to continue with the survey. Otherwise, you will not be able to participate in this study. Thank you.]*
- *If ‘I do not wish to continue this survey’ is selected, the survey is terminated.*

## GENERAL INSTRUCTIONS FOR COMPLETING THE QUESTIONNAIRE

- The questionnaire has 4 sections:
  - Section 1: Personal Background (Anonymous)
  - Section 2: Current Situation
  - Section 3: Cognitive Health
  - Section 4: Opinion
- We recommend completing the questionnaire in one sitting (please allow 30 minutes)
- Questions with response options consisting of circles (○) allow only one answer and you need to select the single answer that best reflects your situation.
- Questions with response options consisting of square boxes (□) allow multiple answers and you can select all the answers that you consider to be true.

|  | SECTION 1: PERSONAL BACKGROUND (ANONYMOUS) |
| --- | --- |
|  | **In which country do you live?** (Please select one answer)   - Belgium - China - Denmark - Finland - France - Germany - Greece - Italy - The Netherlands - Norway - Portugal - Spain - Sweden - None of these 🡪 (END - Acknowledgement and closing) |
|  | **Have you been diagnosed by a doctor**, or do you have a **close relative diagnosed by a doctor**, with any of the following medical conditions? (close relative means a parent, brother, sister, or child) (Please select all that apply)   - Major Depressive Disorder - Post-Traumatic Stress Disorder - Attention-Deficit/Hyperactivity Disorder - Bipolar Disorder - Schizophrenia (ALL RESPONDENTS SHOULD TICK, AT LEAST, THIS ONE, IF NOT 🡪 END - Acknowledgement and closing) - Other (please tell us which condition:_____)[text field] |
|  | This questionnaire is aimed at people who have been diagnosed with schizophrenia and relatives/friends of people living with schizophrenia.  (Please select one answer)  **Please indicate that if you are.**   - A person living with schizophrenia (CODE 1) - Relative/friend/neighbour of a person living with schizophrenia (CODE 2) - None of these 🡪 (END - Acknowledgement and closing) |
|  | [PROGRAMMING: This question is only for caregivers, CODE 2 in Q3] **What is your relationship to the person living with schizophrenia? You are their…**   - Mother/Father - Daughter/Son - Sister/Brother - Wife/Husband - Other relative (please tell us which relative:_____)[text field] - Close friend or neighbour - None of these 🡪 (END - Acknowledgement and closing) |
|  | **How old are you?**  (Please select one answer)   - UNDER 18 years old 🡪 (END - Acknowledgement and closing) - 18-24 years old - 25-34 years old - 35-44 years old - 45-54 years old - 55-64 years old - 65-74 years old - ABOVE 74 years old |
|  | Please, indicate **your gender**:  (Please select one answer)   - Male - Female - Other - Rather not say |
|  | What is the **highest level of education you have finished**?  (Please select one answer)   - Primary education (primary school) - Secondary education (up to, but not including, university) - University: bachelor degree (or equivalent) - University: master degree (or equivalent) - University: PhD or medical degree (or equivalent) - Other (please tell us:____)[text field] |
|  | What is your **current working situation**? Please select the option that best describes your current job status.  (Please select one answer)   - Employed full-time - Employed part-time - Employed but on long term sick leave - Self-employed or freelance - Unemployed, actively looking for a job - Unemployed, not actively looking for a job - Unemployed, not actively looking for a job because of long-term sickness - Student (not employed) - Homemaker (not employed outside of the home) - Doing occupational therapy or a similar assisted work scheme - Doing a program in the community (like a program that helps people meet and join in community events) - Volunteering full-time - Volunteering part-time - Retired at official state retirement age - Retired earlier than the official state retirement age, because of disability/sickness - Retired earlier than the official state retirement age, for another reason - Other (please tell us: ______)[text field] |
|  | **QUESTIONS FOR PATIENTS**  **[PROGRAMING, Q9 to Q12 only for PATIENTS, CODE 1 in Q3]** |
|  | Which of the following answers best describes your current living arrangement?  (Please select one answer)   - Living alone without social support - Living alone with social support - Living with parent / parents - Living with other family member - Living with partner/wife/husband/spouse - Living with friend / friends - Living in an institution - Living in a care home - Living in an assisted living facility - Living in supported accommodation - Other (please tell us:____)[text field] |
|  | At what age were you first diagnosed with schizophrenia?  ___________________years old [Range 0-99] |
|  | Do you currently experience any of the following situations? (Please select all that apply)   - Challenges getting or keeping a job - Challenges with schoolwork or your education - Challenges finishing school - Challenges keeping a steady place to live - Overuse of drugs or alcohol - Being treated unfairly or judged because of something (e.g., stigma and discrimination) - Challenges with friends (please tell us:_____)[Text field] - Challenges to start a family - None of these |
|  | Do you have, or have you had, any of these conditions? (Please select all that apply)   - Cancer (which type of cancer?:______) [Text field] - Diabetes - Obesity - High blood pressure (hypertension) - Heart problems e.g. angina, heart attack - Stroke or mini-stroke - Blood vessel problems / artery diseases e.g. furring of the arteries, blood clots - Sleep problems e.g. insomnia - More frequent infections than most people, e.g. ‘flu, pneumonia, etc - Alcohol misuse disorder - Drug misuse disorder - Anxiety, panic disorder, or other anxiety-type condition - Depression - Other mental health problem (which type?:_____) [Text field] - None of these |

|  | QUESTIONS TO CAREGIVERS  PROGRAMMING, Q13 to Q19 only for CAREGIVERS, CODE 2 in Q3 |
| --- | --- |
|  | At what age was your relative/friend first diagnosed with schizophrenia?  ____________ [Range 0-99] |
|  | Related to your relative/friend with schizophrenia, please indicate which of the following answers best describes their current living arrangement?  (Please select one answer)   - Living alone without social support - Living alone with social support - Living with parent / parents - Living with other family member - Living with partner/wife/husband/spouse - Living with friend / friends - Living in an institution - Living in a care home - Living in an assisted living facility - Living in supported accommodation - Other (please tell us:____) [Text field] |
|  | Related to your relative/friend with schizophrenia, please indicate their current working situation.  (Please select one answer)   - Employed full-time - Employed part-time - Employed but on long term sick leave - Self-employed or freelance - Unemployed, actively looking for a job - Unemployed, not actively looking for a job - Unemployed, not actively looking for a job because of long-term sickness - Student (not employed) - Homemaker (not employed outside of the home) - Doing occupational therapy or a similar assisted work scheme - Doing a program in the community (like a program that helps people meet and join in community events) - Volunteering full-time - Volunteering part-time - Retired at official state retirement age - Retired earlier than the official state retirement age, because of disability/sickness - Retired earlier than the official state retirement age, for another reason - Other (please tell us: ______)[Text field] |
|  | Since they were diagnosed with schizophrenia, how many times have they gone through a period (of at least 2 weeks’ duration) where their psychosis got significantly worse?  (Please select one answer)   - Never - Once or twice - 3 to 5 times - 6 to 10 times - More than 10 times - I don’t know/don’t remember |
|  | Are **they** currently experiencing any of the following situations? (Please select all that apply)   - Challenges getting or keeping a job - Challenges with schoolwork or your education - Challenges finishing school - Challenges keeping a steady place to live - Overuse of drugs or alcohol - Being treated unfairly or judged because of something (e.g., stigma and discrimination) - Challenges with friends (please tell us:_____) [Text field] - Challenges to start a family - None of these |
|  | Related to your relative’s/friend’s schizophrenia, have they ever confided in you that they have experienced thoughts of ending their live? (Please select one answer)   - Yes - No - I don’t know |
|  | Do they have, or have they had, any other condition? (Please select all that apply)   - Cancer (which type?:______) [Text field] - Diabetes - Obesity - High blood pressure (hypertension) - Heart problems e.g. angina, heart attack - Stroke or mini-stroke - Blood vessel problems / artery diseases e.g. furring of the arteries, blood clots - Sleep problems e.g. insomnia - More frequent infections than most people, e.g. pneumonia, ‘flu, etc - Alcohol misuse disorder - Drug misuse disorder - Anxiety, panic disorder, or other anxiety-type condition - Depression - Other mental health problem (which type?:_____) [Text field] - None of these |

**SECTION 2: CURRENT SITUATION**

We would like to ask you about your experience **from when you were diagnosed with schizophrenia** **to the present moment**. We will go step by step asking you some questions about key moments.

|  | **DIAGNOSIS (for patients)**  **[PROGRAMMING Q20 to Q23 only for PATIENTS, CODE 1 in Q3]** |  |
| --- | --- | --- |
|  | Before your current diagnosis was confirmed, **had you ever heard about schizophrenia**?  (Please select one answer)   - Yes - No - I’m not sure |  |
|  | How did you **feel after you were diagnosed with schizophrenia**? Please answer each question thinking about how you felt back then, not how you feel right now.  (Please select all that apply)   - Scared - Relieved - Calm - Concerned - Confused - Angry - Worried - Depressed - Anxious - Sad - Upset - Nothing / not much - I didn’t agree with the diagnosis - I don’t remember |  |
|  | **Right after your diagnosis**, what were you told by your healthcare professional(s)? (Please select all that apply)   - Information about schizophrenia - Information about treatments available - Information about what usually happens as schizophrenia goes on over time - Information about possible side effects of treatments - Useful contacts for getting help - Useful resources like guides or websites for patients - None of this - I don’t remember |  |
|  | **Right after your diagnosis,** who told you information about the condition? (Please select all that apply)   - Psychiatrist - Psychologist - Psychotherapist - Specialist nurse - Community nurse / regular nurse - General Practitioner - Occupational therapist - Social Worker - Case handler - Specialist staff at my place of employment - Patient Advocacy Group / Peer Support Group - Other person with schizophrenia - My caregiver, friend or family member - Others. (Please tell us who:___________)[Text field] |  |
|  | **CURRENT STATE OF HEALTH (for patients)**  **[PROGRAMMING Q24 to Q37 only for PATIENTS, CODE 1 in Q3]** | |
|  | We want to ask you some questions about how you're doing each day with your illness and your current health. | |
|  | Who do you usually see to talk about schizophrenia? (Please select all that apply)   - Psychiatrist - Psychologist - Psychotherapist - Specialist nurse - Community nurse / regular nurse - General Practitioner - Occupational therapist - Social Worker - Case handler - Specialist staff at my place of employment - Patient Advocacy Group / Peer Support Group - Other person with schizophrenia - My caregiver, friend or family member - Others. (Please tell us who:___________) [Text field] | |
|  | How often do you typically meet your [PROGRAMMING: Trigger this question for each of the answers selected in Q24]  (Please select only one answer)   - Every week - Every two weeks - Once a month - Every 2 months - Every 3 months - Every 4 months - Every 6 months - Once a year - Rarely/On special occasions - Never - Other (please tell us:________)[Text field] | |
|  | At present, how often would you say with regards to schizophrenia…   \|  \| None of the time \| A little of the time \| Some of the time \| Most of the time \| All of the time \| Not Applicable \| \| --- \| --- \| --- \| --- \| --- \| --- \| --- \| \| I get support from my family \| o \| o \| o \| o \| o \| o \| \| I get support from my friend(s) \| o \| o \| o \| o \| o \| o \| \| I get support from my neighbour(s) \| o \| o \| o \| o \| o \| o \| \| I get support from a healthcare professional at home \| o \| o \| o \| o \| o \| o \| \| I get support from a social worker at home \| o \| o \| o \| o \| o \| o \| \| I get financial support from family / friends (money) \| o \| o \| o \| o \| o \| o \| \| I get psychological support from a professional \| o \| o \| o \| o \| o \| o \| \| I get help from someone with schizophrenia or a patients’ group \| o \| o \| o \| o \| o \| o \| \| I get help from my religious or spiritual beliefs \| o \| o \| o \| o \| o \| o \| \| The state / national health insurance gives me free-of-charge medications \| o \| o \| o \| o \| o \| o \| \| The state / national health insurance gives me less expensive medications \| o \| o \| o \| o \| o \| o \| \| The state / national health insurance gives me free-of-charge in-person therapy \| o \| o \| o \| o \| o \| o \| \| The state / national health insurance gives me less expensive in‑person therapy \| o \| o \| o \| o \| o \| o \| | |
|  | Where **do you get most of the information/support about the condition**?  (Please select all that apply)   - From my specialist doctor e.g., psychiatrist - From my psychologist - From my psychotherapist - From my specialist nurse - From my community nurse / regular nurse - From my General Practitioner - From my occupational therapist - From specialist staff at my place of work - From my social worker - From my case handler - From my pharmacist - From the internet - From social media - From a patient’s association/society/support group (please tell us what the group is called: ____) [Text field] - From research papers and/or scientific websites - From patient support tools (e.g., patient guides to support you, patient websites…) - From another person with schizophrenia - My caregiver, friend or family member - From my romantic partner, spouse, family member, professional caregiver, etc. - Other (please tell us which: _____)[Text field] - I don’t get information - I don’t know | |
|  | Which treatment do you take for the condition at the moment? (Please select all that apply)   - Depot antipsychotic medication (given by injection) (e.g. depixol, fluanxol, haloperidol, risperidone, olanzapine, aripiprazole) - Oral antipsychotic medication (tablets/capsules that you swallow) (e.g. aripiprazole, olanzapine, quetiapine, clozapine, risperidone, haloperidol) - Mood stabilizers (e.g. lithium, valproate, carbamazepine, lamotrigine) - Antidepressant medication (e.g. fluoxetine, sertraline, citalopram, escitalopram, venlafaxine, duloxetine mirtazapine) - Anti-anxiety medication (e.g. lorazepam, diazepam, clonazepam, buspirone, propranolol, pregabalin) - Sleep medication (e.g. promethazine, zopiclone) - Other: (please tell us the names of the medications______)[Text field] - I don’t know - None of these - I don’t take any medications at all | |
|  | Have you had any of the following treatments for schizophrenia, in the past or currently?  (Please select all that apply)   - Cognitive Remediation Therapy (CRT): e.g. exercises and games to train your thinking skills, memory, and problem-solving abilities. - Cognitive Behavioural Therapy (CBT): e.g. talking with a therapist to identify and challenge negative thoughts and behaviours and find healthier ways to deal with them. - Occupational Therapy (OT): e.g. practicing daily tasks like cooking, dressing, writing, or something else, with the guidance of a therapist. - Eye Movement Desensitization and Reprocessing (EMDR): e.g. you talk through traumatic memories while moving your eyes, or holding buzzers, or tapping your hands. - Social Skills Therapy: e.g. learning and practicing communication and social interactions. - Training in Mindfulness or Breathing Techniques: e.g. learning and practicing deep breathing, meditation, mindfulness, etc. - Psychoanalysis: e.g. a psychotherapist asking you questions about your past. - Music Therapy: e.g. using music to express and understand your emotions, with a therapist. - Interdisciplinary Team Support: coordinated care from a team including various health professionals to manage treatment outside of the hospital. - None of these - I don’t know / I don’t remember | |
|  | Did you have to stop school, education and/or working because of schizophrenia?   - Yes - No - I don’t know | |
|  | Did you have to change your job to an easier one because of the condition?   - Yes - No - I don’t know | |
|  | How often do you now feel less productive, compared to before you were diagnosed with the condition?   - Never - Rarely - Sometimes - Often - Very often | |
|  | Do you currently receive any help, benefits or payments from the government or state programs because of schizophrenia?  (Please select all answers that you think apply)   - No - Supported living - Pension because of schizophrenia - Income support - Pharmaceutical assistance (medicines free of charge or at a lower cost) - Rehabilitation assistance (rehab treatment free of charge or at a lower cost) - Public transport discount - Access to discounted holidays | |
|  | Do you receive practical help for your day-to-day activities from any of the following people/services? If yes, how difficult or easy is it/was it to get this help?   \|  \| Very difficult \| Difficult \| Okay \| Easy \| Very easy \| Not Applicable \| \| --- \| --- \| --- \| --- \| --- \| --- \| --- \| \| Family \| O \| O \| O \| O \| O \| O \| \| Friends \| O \| O \| O \| O \| O \| O \| \| Neighbours \| O \| O \| O \| O \| O \| O \| \| Local social services \| O \| O \| O \| O \| O \| O \| \| State social services \| O \| O \| O \| O \| O \| O \| \| Employer \| O \| O \| O \| O \| O \| O \| | |
|  | Thinking about how you are doing right now, please tell us how much you agree with the following…   \|  \| Don’t agree at all \| Don’t really agree \| Indifferent \| Slightly agree \| Strongly agree \| Don’t know \| \| --- \| --- \| --- \| --- \| --- \| --- \| --- \| \| I don’t have schizophrenia \| O \| O \| O \| O \| O \| O \| \| Overall, my schizophrenia is perfectly under control and manageable \| O \| O \| O \| O \| O \| O \| \| I understand my schizophrenia well enough \| O \| O \| O \| O \| O \| O \| \| I can handle my medical care \| O \| O \| O \| O \| O \| O \| \| I can spot issues linked to my schizophrenia \| O \| O \| O \| O \| O \| O \| \| I can handle challenges related to my schizophrenia \| O \| O \| O \| O \| O \| O \| \| I can deal with any side effects of my schizophrenia medication \| O \| O \| O \| O \| O \| O \| | |
|  | What type of food to you eat **more often than any other type of food?** (Please select one answer)   - Food I cook myself from fresh ingredients - Food someone else cooks for me from fresh ingredients (Please tell us who cooks it: ___) [Text field] - Eat out, e.g. at restaurants - Ready-meals bought at the shop - Snacks bought at the shop (e.g. sandwiches, chips, peanuts, etc.) - Take-aways | |
|  | Do you do any of the following? (Please select all that apply)   - Shower or bathe at least every other day - Brush teeth two times a day or more - Always wash your hands before you eat - Always wash your hands after you go to the toilet - Use something every day to stop your sweat smelling e.g. deodorant, antiperspirant - Cut your nails often to keep them short - Put on clean clothes at least every other day - Clean where you live every week - Use sunblock when you’re outside for a long time | |

**SECTION 3: COGNITIVE HEALTH**

Now, we would like to focus on cognitive health (thinking capabilities) sometimes affected by schizophrenia.

|  | **KNOWLEDGE** |
| --- | --- |
|  | [PROGRAMMING: For PATIENTS only, CODE 1 in Q3]  Some people have symptoms that affect how they think and function. Have you ever noticed the following symptoms?  (Please select all that apply)   - Memory problems, (e.g. hard to remember something long enough to make decisions) - Hard to stay focused on tasks - Hard to plan or solve everyday problems - Hard to remember spoken instructions (e.g. if someone told you to do something, you often forget) - Hard to remember things or people you saw before (e.g. not recognising people you already met many times) - Slowness in thinking - Hard to understand how other people feel, or what they mean, in social situations - Hard to get people to understand what you are saying, or to understand what other people mean - None of these |
|  | [PROGRAMMING: For CAREGIVERS only, CODE 2 in Q3]  Many people have symptoms that affect how they think and function. Have you ever noticed the following symptoms in the person you care for?  (Please select all that apply)   - Memory problems, (e.g. difficult to remember something long enough to make decisions) - Trouble to stay focused on tasks - Trouble to plan or solve everyday problems - Trouble to remember spoken instructions (e.g. if someone told you to do something, you often forget) - Trouble to remember things or people you saw before (e.g. not recognising people you already met many times) - Slowness in thinking - Trouble to understand how other people feel, or what they mean, in social situations - Trouble to get people to understand what you are saying, or to understand what other people mean - None of these |
|  | Have you ever **heard about “cognitive impairment associated with schizophrenia”?**  (Please select one answer)   - Yes CODE 1 - No CODE 2 - I am not sure CODE 3 - I don’t remember CODE 4 |
|  | [PROGRAMMING: Only for those ticked YES, CODE 1 in Q40]  **Where/from whom** have you heard about “cognitive impairment associated with schizophrenia”?  (Please select all that apply)   - From a psychiatrist - From a psychologist / psychotherapist - From a specialist nurse - From a General Practitioner (GP) - From the pharmacist - From a romantic partner, spouse, family member, professional caregiver, etc. - From someone else who has been diagnosed with schizophrenia - From the internet - From social media - From a television program, documentary or news report - From brochures or leaflets - From a course - From patient’s association/society/patient support group - From research papers and/or scientific websites - I don’t know |
|  | **MONITORING & TREATMENT**  **[PROGRAMMING Q42 to Q46 only for PATIENTS, CODE 1 in Q3]** |
|  | Do the healthcare professionals you meet ever ask you about your cognitive performance, memory or ability to solve problems?  (Please select one answer)   - Never [GO to Q46] - Rarely - Sometimes - Often - Very often - I do not know [GO to Q46] |
|  | Which professionals **ask you about your cognitive performance, memory or ability to solve problems**? (Please select all that apply)   - Psychiatrist - Psychologist - Psychotherapist - Specialist Nurse - Community nurse - General Practitioner - Social Worker - Others - None of these |
|  | Do the healthcare professionals you meet ever do any **tests or questionnaires** to test your brain’s abilities, memory or ability to solve problems?  (Please select one answer)   - Never [GO to Q46] - Rarely - Sometimes - Often - Very often - I do not know [GO to Q46] |
|  | Which professionals perform **tests or questionnaires** to test your brain’s abilities, memory or ability to solve problems? (Please select all that apply)   - Psychiatrist - Psychologist - Psychotherapist - Specialist Nurse - Community nurse - General Practitioner - Social Worker - Others - None of these |
|  | **Have you received any support or specific treatment** (medication or in-person therapy) specific for cognitive impairment?  (Please select one answer)   - Yes, I’m currently getting it (Please tell us which type:_____)[Text field] - Yes, in the past but not right now (Please tell us which type:_____) [Text field] - No, never - I do not know |
|  | **BURDEN**  **[PROGRAMMING Q47 to Q51 only for PATIENTS, CODE 1 in Q3]** |
|  | Please tell us how much you worry or care that you have the following problems. Please rate your answers from 0 to 5. 0 means you don’t care/worry at all, 5 means it is a very important worry.   \| [PROGRAMMING: only show rows that respondents selected in Q38] \| **0**  **Don’t care at all** \| **1** \| **2** \| **3 Medium** \| **4** \| **5**  **A very important worry** \| **Don’t know / not applicable** \| \| --- \| --- \| --- \| --- \| --- \| --- \| --- \| --- \| \| 1. Memory problems, (e.g. hard to remember something long enough to make decisions) \| O \| O \| O \| O \| O \| O \| O \| \| 1. Hard to stay focused on tasks \| O \| O \| O \| O \| O \| O \| O \| \| 1. Hard to plan or solve everyday problems \| O \| O \| O \| O \| O \| O \| O \| \| 1. Hard to remember spoken instructions (e.g. if someone told you to do something, you often forget) \| O \| O \| O \| O \| O \| O \| O \| \| 1. Hard to remember things or people you saw before (e.g. not recognising people you already met many times) \| O \| O \| O \| O \| O \| O \| O \| \| 1. Slowness in thinking \| O \| O \| O \| O \| O \| O \| O \| \| 1. Hard to understand how other people feel, or what they mean, in social situations \| O \| O \| O \| O \| O \| O \| O \| \| 1. Hard to get people to understand what you are saying, or to understand what other people mean \| O \| O \| O \| O \| O \| O \| O \| |
|  | Do you think problems with your memory, ability to pay attention, concentration and/or planning have led you to lose valuable friendships?   - Yes - No |
|  | Do you think problems with your memory, ability to pay attention, concentration and/or planning have had a bad impact on your relationships with family members, friends or colleagues?   - Yes - No |
|  | Thinking about your relationships with other people, do you agree with any of the following statements?   \|  \| YES \| NO \| \| --- \| --- \| --- \| \| People assume I don’t join in conversations because I’m not interested, but it’s really because I’m finding it hard to keep up \| O \| O \| \| When I forget details or appointments, others think I’m being careless or not paying attention. \| O \| O \| \| People get annoyed with me for asking repetitive questions, but it’s really because I struggle remembering things. \| O \| O \| \| People get annoyed with me when I need things to be explained several times, seeing it as me being stubborn. \| O \| O \| \| People misunderstand my need to write things down to remember, as a lack of effort for me to remember things on my own. \| O \| O \| \| People think I’m not trying to understand them, when really I’m trying but finding it difficult. \| O \| O \| \| It is hurtful when people joke about my ‘forgetfulness’. \| O \| O \| \| I often feel lonely. \| O \| O \| |
|  | **How much support** do you need from caregivers relatives or friends, to do the following tasks? Please rate your answers from 0 to 5. 0 means no support at all, 5 means you can’t do the task alone.   \| **Support needed** \| **0**  **No support needed** \| **1** \| **2** \| **3 Medium** \| **4** \| **5**  **Cannot do the task alone** \| **Don’t know / not applicable** \| \| --- \| --- \| --- \| --- \| --- \| --- \| --- \| --- \| \| 1. Buying groceries \| O \| O \| O \| O \| O \| O \| O \| \| 1. Preparing meals \| O \| O \| O \| O \| O \| O \| O \| \| 1. Managing finances \| O \| O \| O \| O \| O \| O \| O \| \| 1. Scheduling and keeping appointments \| O \| O \| O \| O \| O \| O \| O \| \| 1. Organizing and remembering to take medications \| O \| O \| O \| O \| O \| O \| O \| \| 1. Cleaning and organizing my home \| O \| O \| O \| O \| O \| O \| O \| \| 1. Natigating public transport or driving \| O \| O \| O \| O \| O \| O \| O \| \| 1. Doing well in my studies or at work \| O \| O \| O \| O \| O \| O \| O \| \| 1. Communicating well with my healthcare providers \| O \| O \| O \| O \| O \| O \| O \| \| 1. Asking for help from professionals (doctors, pharmacists, etc.) \| o \| o \| o \| o \| o \| o \| o \| |
|  | **BURDEN**  **[PROGRAMMING Q52 to Q64 only for CAREGIVERS, CODE 2 in Q3]** |
|  | **How much support** does the person you care for need from caregivers, relatives or friends, to do the following day-to-day activities? Please rate your answers from 0 to 5. 0 means no support at all, 5 means they cannot do the task alone.   \| **Support needed** \| **0**  **No support needed** \| **1** \| **2** \| **3 Medium** \| **4** \| **5**  **Cannot do the task alone** \| **Don’t know / not applicable** \| \| --- \| --- \| --- \| --- \| --- \| --- \| --- \| --- \| \| 1. Buying groceries \| O \| O \| O \| O \| O \| O \| O \| \| 1. Preparing meals \| O \| O \| O \| O \| O \| O \| O \| \| 1. Managing finances \| O \| O \| O \| O \| O \| O \| O \| \| 1. Scheduling and keeping appointments \| O \| O \| O \| O \| O \| O \| O \| \| 1. Organizing and remembering to take medications \| O \| O \| O \| O \| O \| O \| O \| \| 1. Cleaning and organizing their home \| O \| O \| O \| O \| O \| O \| O \| \| 1. Navigating public transport or driving \| O \| O \| O \| O \| O \| O \| O \| \| 1. Doing well in their studies or at work \| O \| O \| O \| O \| O \| O \| O \| \| 1. Communicating effectively with their healthcare providers \| O \| O \| O \| O \| O \| O \| O \| \| 1. Asking for help from professionals (doctors, pharmacists, etc.) \| O \| O \| O \| O \| O \| O \| O \| |
|  | How much time do you spend each week caring for your relative/friend with schizophrenia?  ___________hours [Range 0-168] |
|  | Do you or your relative/friend currently receive any of the following support from the state or charities?  (Please select all that apply)   - Financial support - Retirement and pension because of my caregiving role - Free-of-charge caregiving support for my relative/friend - Free-of-charge medicines for my relative/friend - Rehabilitation assistance for my relative/friend (free-of-charge or discounted) - Access to welfare jobs / supported employment programs - Free-of-charge or discounted holidays or rest retreats for my relative/friend - Free-of-charge or discounted holidays or rest retreats for me - Public transport discount for my relative/friend - Other (Please tell us:______)[Text field] - None of these |
|  | Regarding caring for your relative/friend with schizophrenia, do you wish you had more support from the state or charities?   - Yes CODE 1 - No CODE 2 |
|  | (PROGRAMMING: Only if answered YES, CODE 1 in Q56) What type of support would you welcome? (Please select all that apply)   - Financial support - Retirement and pension because of my caregiving role - Free-of-charge caregiving support for my relative/friend - Free-of-charge medicines for my relative/friend - Rehabilitation assistance for my relative/friend (free-of-charge or discounted) - Access to welfare jobs / supported employment programs - Free-of-charge or discounted holidays or rest retreats for my relative/friend - Free-of-charge or discounted holidays or rest retreats for me - Public transport discount for my relative/friend - Other (Please tell us:______)[Text field] - None of these |
|  | Has caring for your relative/friend with schizophrenia affected your work in any of the following ways? (Please select all that apply)   - I have chosen to reduce hours at my employed job (How many hours per week did you reduce by?: ____hours) [Range: 0-120] - I have been forced to reduce hours at my employed job (How many hours per week did you reduce by?: ____hours) [Range: 0-120] - I have been fired from my job - I have chosen to quit my career - My career progression has slowed down - I have lost interest in my career - I used to volunteer for charity but now I don’t - I would have linked to volunteer for charity but now I can’t - None of these |
|  | Has caring for your relative/friend with schizophrenia affected your social life in any of the following ways? (Please select all that apply)   - I have lost valuable friendships - I have gained new valuable friendships - I don’t socialise as much as I’d like to - My friends don’t understand what I’m going through - I often feel lonely / isolated - None of these |
|  | Has caring for your relative/friend with schizophrenia affected your mental health in any of the following ways? (Please select all that apply)   - I am often anxious - I am often stressed - I often feel overwhelmed - I often feel hopeless - I have been diagnosed with an anxiety disorder - I have been diagnosed with depression - I have been diagnosed with a stress disorder / post-traumatic stress disorder (PTSD) - I have needed support from professionals for my own mental health - I have lost my purpose in life - Caring for my relative/friend with schizophrenia gives me a greater purpose in life - None of these |
|  | Some people describe their relative/friend with schizophrenia in the following ways. Would you also describe them in these ways? (Please select all that apply)   - They are lazier than they used to be - They go against me on purpose - They don’t try hard enough to do their daily tasks - They have emotional outbursts for no reason - They are like a shell of their former self - Caring for them is like caring for a perpetual child - They often seem to have their head in the clouds - They are not interested to join in on conversation - They are often careless or don’t pay attention, so forget important details or appointments - It often frustrates me that they ask repetitive questions - They are often stubborn, asking a simple concept to be explained several times. - They don’t make enough effort to remember things - They don’t make enough effort to understand me - None of these |
|  | Do you feel you have lost hope that your relative/friend will get better?   - Yes - No - I don’t know |
|  | Has your relative/friend ever been homeless because of their schizophrenia?   - Yes - No |
|  | Do you think society loses out on the skills that **your relative/friend** might bring, because they don’t get the right help and support they need?   - Yes - No |
|  | Do you think society loses out on the skills that **you** might bring, because of your caregiving responsibilities?   - Yes - No |

**SECTION 4: OPINION**

|  | **DEGREE OF AGREEMENT** |
| --- | --- |
|  | Lastly, based on your knowledge and experience, please state your degree of agreement with the following statements related to the treatment of cognitive impairment associated with schizophrenia, being 1 completely disagreeing and 9 completely agreeing: (Please select one answer per row) |

|  | **1**  **Completely**  **disagree** | **2** | **3** | **4** | **5** | **6** | **7** | **8** | **9**  **Completely**  **agree** | **I do not know** |
| --- | --- | --- | --- | --- | --- | --- | --- | --- | --- | --- |
| 1. Cognitive symptoms are a core feature of schizophrenia. | O | O | O | O | O | O | O | O | O | O |
| 1. People with schizophrenia and mild cognitive symptoms usually know they have poor cognitive health. (mild: able to maintain daily activities independently) | O | O | O | O | O | O | O | O | O | O |
| 1. People with schizophrenia and moderate-severe cognitive symptoms usually know they have poor cognitive health. (moderate-severe: require support to maintain daily activities) | O | O | O | O | O | O | O | O | O | O |
| 1. People with schizophrenia and cognitive symptoms often blame these cognitive symptoms on side effects from their psychotropic medications. | O | O | O | O | O | O | O | O | O | O |
| 1. All healthcare practitioners treating with people with schizophrenia inform their patients about cognitive symptoms. | O | O | O | O | O | O | O | O | O | O |
| 1. There is a need to increase awareness of cognitive impairment associated with schizophrenia among psychiatric healthcare practitioners. | O | O | O | O | O | O | O | O | O | O |
| 1. There is a need to increase awareness of cognitive impairment associated with schizophrenia among non‑psychiatric healthcare practitioners. | o | o | o | o | o | o | o | o | o | o |
| 1. There is a need to increase awareness of cognitive impairment associated with schizophrenia among people with schizophrenia. | O | O | O | O | O | O | O | O | O | O |
| 1. There is a need to increase awareness of cognitive impairment associated with schizophrenia among relatives/caregivers. | O | O | O | O | O | O | O | O | O | O |
| 1. There is a need to increase awareness of cognitive impairment associated with schizophrenia in society. | O | O | O | O | O | O | O | O | O | O |

Thank you for completing the questionnaire.

Your feedback is very much appreciated.
