# Supplementary material for: Understanding the burden of cognitive impairment associated with schizophrenia: Results from the international LUCIA study
Source: Eur Psychiatry. 2026 Apr 28;69(1):e55. doi: 10.1192/j.eurpsy.2026.12208 (PMC13227134; doi:10.1192/j.eurpsy.2026.12208)
Supplement: Correll et al. supplementary material [file S0924933826122081sup001.zip › SUPPL_METHODS.docx]

Supplementary detailed methods

## Study design and rationale

We conducted a cross‑sectional, multi‑country, multi‑stakeholder study using a three‑phase, Delphi‑informed design to collate and structure expert and experiential perspectives on CIAS. In accordance with the established ACCORD guidelines for conducting and reporting consensus studies (33,34), the design combined exploratory qualitative enquiry with iterative, standardised rating to support transparent consensus building where appropriate, and descriptive mapping where consensus was not the objective.

### Phase 1: Expert opinion

In‑depth individual interviews (IDIs) were conducted with 11 members of the Scientific Committee (SC) to establish the contextual framework, refine the conceptual scope, and generate candidate domains and statements for subsequent enquiry. Insights from this stage informed the sampling frame for later phases and the architecture of the Phase 2 discussion guide and Phase 3 survey instruments.

### Phase 2: Qualitative enquiry

We undertook semi‑structured, remote interviews (≈60 minutes) with 85 participants: 74 health and social care professionals (HCPs) and 11 representatives of caregiver advocacy groups (CAGs) with demonstrable expertise in schizophrenia and CIAS. Interviews were completed between July and October 2023 across 12 countries (Belgium, China, France, Germany, Greece, Italy, Japan, Netherlands, Portugal, Spain, Sweden, United Kingdom) using a structured guide organised into three thematic sections: (1) patient journey and health‑care resource use; (2) humanistic, economic and societal burden; and (3) CIAS management. Thematic outputs were used to refine terminology, prioritise topics, and draft survey items and consensus statements for Phase 3.

### Phase 3: Quantitative Delphi survey

An online survey was deployed to HCPs, patients, and caregivers across 15 countries (multiple European countries plus China and Japan) to further explore the awareness of CIAS, patients’ experiences, and healthcare resources use, and the humanistic, economic and societal burden of the condition. Prior to full implementation, a soft launch was conducted to pilot the questionnaire, ensuring its clarity, comprehensibility, and operational functionality within the intended respondent groups. The questionnaire was translated into local languages. Two tailored versions were implemented (one for HCPs and one for patients/caregivers), with the following content:

- HCP questionnaire: Panellist profile; patient pathway and health‑care resource utilisation; humanistic, economic, and societal burden / unmet needs; CIAS treatment; CIAS‑related burden.
- Patient/Caregiver questionnaire: Panellist profile; current care context; perception of cognitive health; opinions and attitudes.

In order to differentiate between patients with and without CIAS, the HCP questionnaire included the following information:

*Cognitive Impairment Associated with Schizophrenia (CIAS) affects the following domains, in a global manner:*

- *Attention/vigilance (e.g. struggle to read a book),*
- *Working memory (e.g. struggle to remember a phone number just given to you),*
- *Verbal learning and memory (e.g. remembering the items someone told you to buy at the supermarket),*
- *Visual learning and memory (e.g. remembering where you put something in a closet),*
- *Reasoning and problem solving (e.g. arriving on time for work even when the bus schedule has changed),*
- *Speed of processing (e.g. using a touch-screen computer to serve customers at a fast-food restaurant), and*
- *Social cognition (e.g. knowing by looking at someone whether they are angry at you or not).*

*Cognitive symptoms differ from the negative symptoms of schizophrenia which mainly include blunted affect, alogia (lack of speech), reduced motivation and anhedonia (reduced experience of pleasure).*

*CIAS also differs from dementia, which comprises symptoms that are often restricted to the domains of memory.*

As for the assessment in the patient/caregiver questionnaire, CIAS is only specifically mentioned when asking whether they have heard of the terminology and by whom. To determine their cognitive status and impact, general terms such as “cognitive performance, memory, or ability to solve problems” are used.

The complete versions of the questionnaires are provided as supplementary materials 1 and 2, respectively. Item formats included single‑choice, multiple‑choice, open‑ended responses, and consensus statements rated on a 9‑point Likert‑type scale (1 = strongly disagree; 9 = strongly agree).

#### Participants and eligibility

A target sample of up to 821 panellists was prespecified. HCP inclusion required ≥5 years’ experience with schizophrenia, a minimum caseload in the past year (thresholds varied by profession), and ≥40% of patients with clinically evident cognitive impairment. HCP profiles included psychiatrists, psychologists/psychotherapists, specialist nurses, social workers/occupational therapists, and hospital pharmacists. In addition, the panel incorporated adult patients with a diagnosis of schizophrenia and caregivers/relatives of individuals diagnosed with the condition.

#### Delphi Administration

Delphi administration” paragraph: (i) survey platform, (ii) anonymity to peers and to facilitators, (iii) feedback content/format between rounds, (iv) pre-specified number of rounds and conditions for stopping, (v) data storage.

## Outcomes and consensus definition

The survey explored awareness of CIAS, patient pathways and health‑care resource utilisation, humanistic/economic/societal burden, treatment patterns, and unmet needs. For interpretive parsimony, Likert responses were grouped as disagreement (1–3), neutral (4–6), or agreement (7–9). A priori, consensus (agreement or disagreement) was defined as ≥70% of respondents selecting values within the same category. Items failing to meet this threshold in wave 1 were reconsidered in wave 2 (HCPs only), preserving the iterative component characteristic of Delphi approaches.

## Analysis

Analyses were primarily descriptive. Categorical variables are presented as frequencies/percentages, while continuous variables as summary statistics. For consensus statements, category distributions and attainment of the ≥70% threshold were tabulated. Limited, pre‑specified exploratory comparisons (e.g., stratification by presence/absence of CIAS) were undertaken using Student’s t-test or chi-square as adequate, to avoid excess multiplicity. Data analysis was performed using IBM SPSS Statistics V.29.

## Ethical considerations

All participants provided informed consent prior to participation. Study procedures were designed to be consistent with ethical principles for research involving human participants and with applicable data‑protection requirements in participating jurisdictions. Formal ethical approval was not required for this study, as it comprised an opinion-based survey. Patient invitations were managed exclusively by the patient advocacy group (PAG), without involvement of healthcare professionals or access to clinical records at any stage of the process.
