# Supplementary material for: Understanding the burden of cognitive impairment associated with schizophrenia: Results from the international LUCIA study
Source: Eur Psychiatry. 2026 Apr 28;69(1):e55. doi: 10.1192/j.eurpsy.2026.12208 (PMC13227134; doi:10.1192/j.eurpsy.2026.12208)
Supplement: Correll et al. supplementary material [file S0924933826122081sup001.zip › SUPPL_RESULTS.docx]

Supplementary Results

# Sample characteristics

## Survey participation by wave


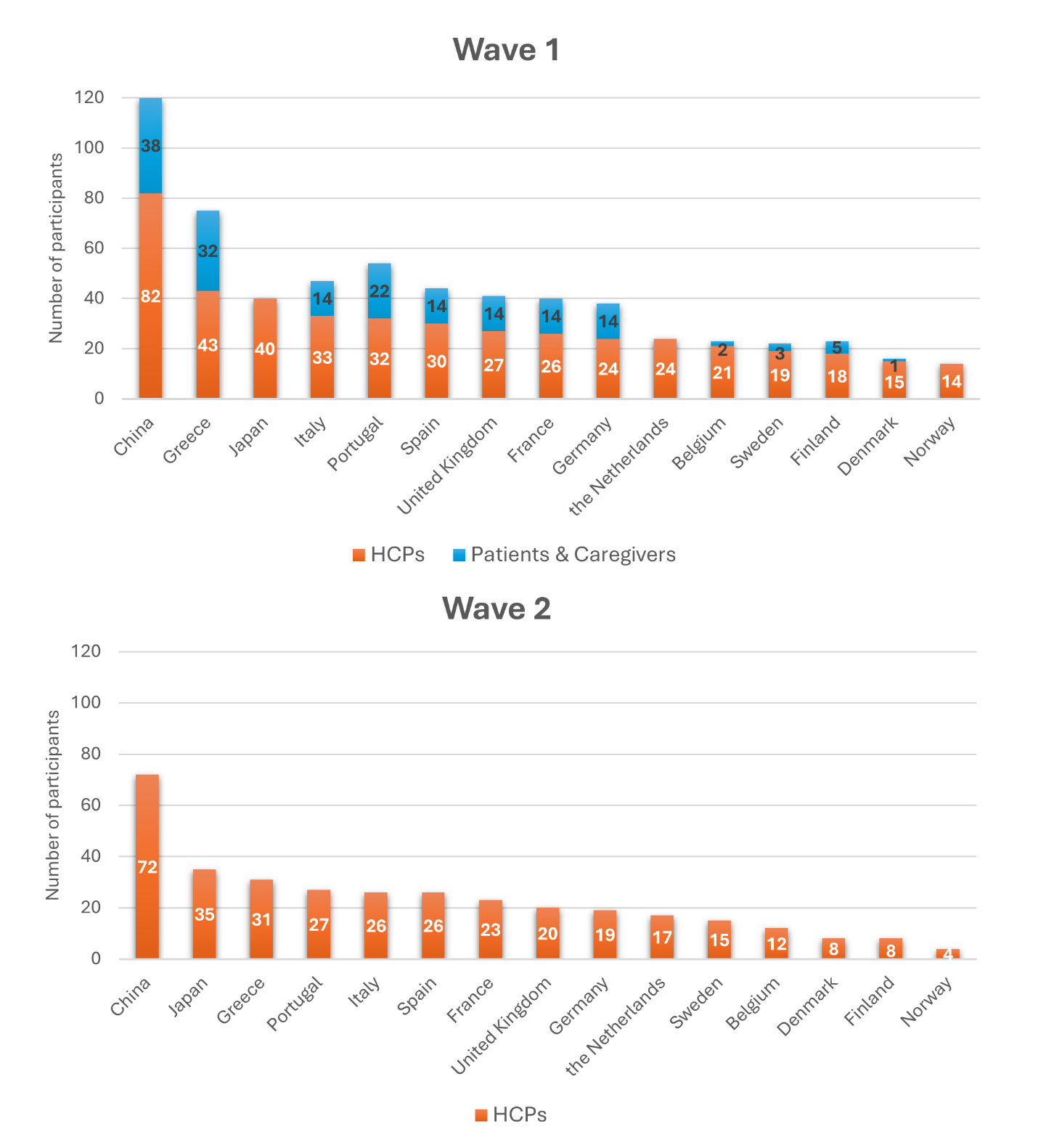


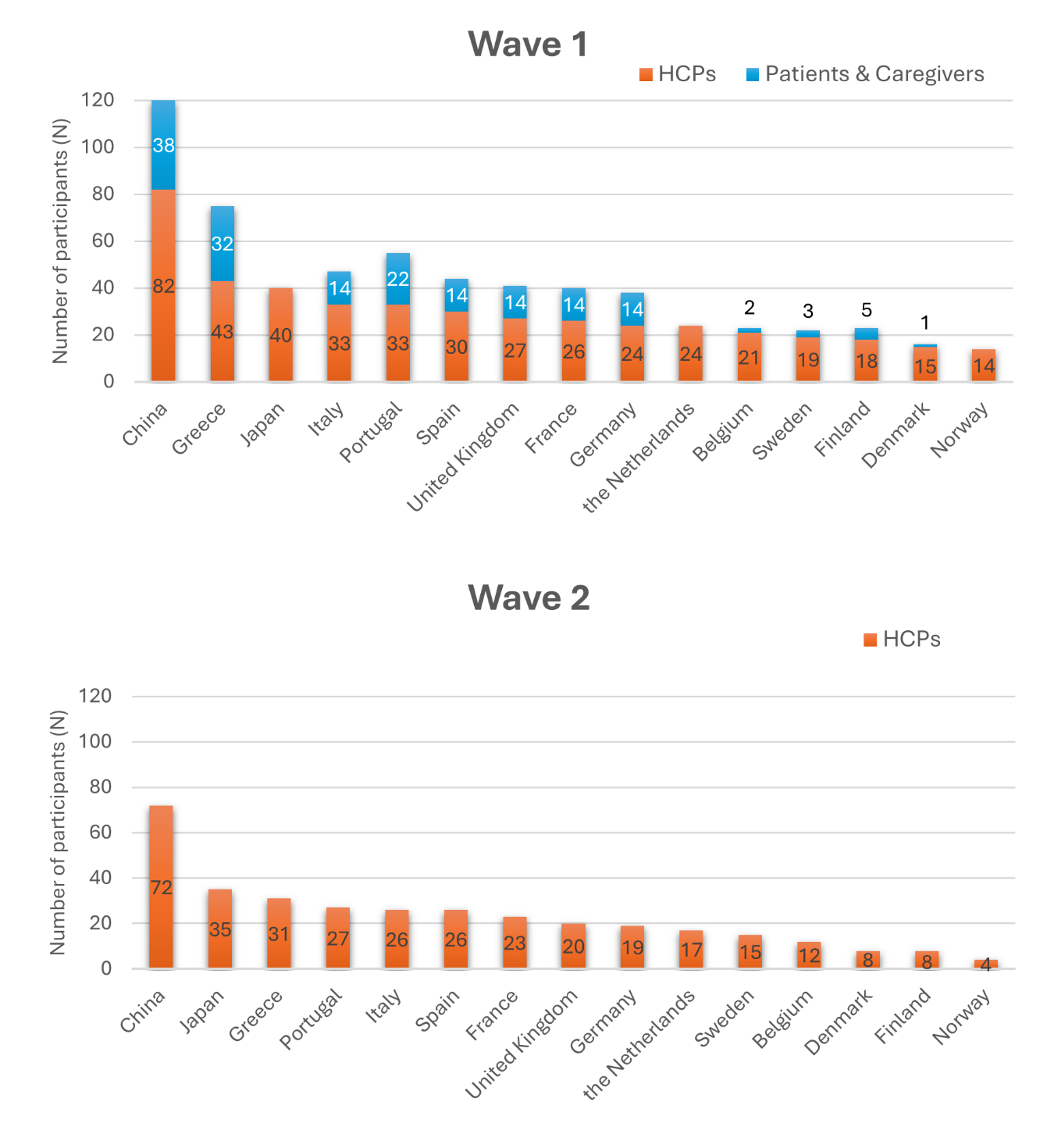


*Supplementary Figure 1. Participation by target type and country, across both waves of the Delphi survey. Abbreviations: HCPs = Health Care Professionals.*

| Targets | *Patients*  *(N=61)* | *Caregivers*  *(N=112)* |
| --- | --- | --- |
| Gender (% females) | *50.8%* | *73.2%* |
| Age (%)  18-24 y  25-34 y  35-44 y  45-54 y  55-64 y  65-74 y  >74 y | *14.8%*  *16.4%*  *36.1%*  *18.0%*  *8.2%*  *4.9%*  *1.6%* | *4.5%*  *17.9%*  *19.6%*  *21.4%*  *23.2%*  *8.9%*  *4.5%* |
| Age at diagnosis (%)  <18  18-24 y  25-34 y  35-44 y  45-54 y  55-64 y  65-74 y  >74 y | *12.0%*  *32.0%*  *28.0%*  *14.0%*  *11.0%*  *0.0%*  *1.0%*  *1.0%* | *N/A* |
| Relationship to CIAS patient (%)  Parent  Child  Sibling  Spouse  Friend/neighbour  Other relative | *N/A* | *32.1%*  *16.1%*  *25.0%*  *16.1%*  *4.5%*  *6.3%* |

Supplementary Table 1. Demographic profiles of patients and caregivers that participated in the study. Abbreviations: CIAS: Cognitive Impairment Associated with Schizophrenia; N: number; N/A: not applicable; y: years.

# CIAS awareness

## Patients and caregivers

Across the SC interviews and qualitative IDIs, experts reported that patients and caregivers are often unaware that CIAS exists or that it is a core feature of schizophrenia. In fact, results from the Delphi survey showed that only 55.7% of patients and 65.2% of caregivers had heard the term ‘cognitive impairment associated with schizophrenia (i.e., CIAS). Although HCPs reached consensus that “cognitive symptoms are a core feature of schizophrenia” (75.9% agreement), patients/caregivers did not (58.9%), indicating gaps in psychoeducation and information transfer.

Routine psychoeducation was inconsistent. For statements that HCPs routinely explain CIAS or warn patients that they may develop cognitive symptoms, complete disagreement was ~19%, complete agreement ~47–49%, with no consensus by HCP type—evidence of heterogeneous practices. Despite the lack of agreements, the reported reasons for lack of psychoeducation in the Delphi survey included limited time (60.2%), lack of guidance (58.1%), insufficient confidence in treatment knowledge (56.5%), and concern about causing fear (55.2%). Most stakeholders endorsed increasing CIAS awareness among relatives and caregivers (patients/caregivers 84.8%; HCPs 83.9%). Among those patients and caregivers familiar with CIAS, psychiatrists were the main information source (68.2%) and were perceived as key educators at diagnosis for schizophrenia (90.1%) and CIAS (75.8%).

## Healthcare professionals

While most psychiatrists recognise CIAS as core, as demonstrated by experts in both the qualitative interviews and Delphi survey, many report difficulty identifying it. Only 52.2% agreed that HCPs have a good understanding of how cognitive symptoms manifest, and 31.5% completely agreed that HCPs are not sufficiently aware that CIAS is part of schizophrenia. There was consensus on the need to raise awareness among psychiatric (78.1%) and non‑psychiatric (79.6%) HCPs and to expand continuing medical education (83.2%). Finally, most patients/caregivers (86.0%) and HCPs (75.8%) endorsed increasing societal awareness.

# CIAS follow-up

Roles and responsibilities for CIAS follow‑up were ill‑defined, with limited coordinated multidisciplinary management. In practice, psychiatrists were the principal follow‑up providers and were commonly seen monthly (38.3%). According to SC interviews, follow‑up frequency depended on severity/progression: in mild CIAS, specific monitoring was typically omitted, whereas severe CIAS prompted targeted follow‑up every 3–6 months. Even when CIAS was identified, formal monitoring was uncommon, as only 15.8% of patients reported that HCPs often/very often investigate cognition. Importantly, experts reported limited social‑care follow‑up, lacking specific tools. Reported barriers included poor awareness of CIAS importance among HCPs, lack of effective treatments, and absence of a freely available, validated monitoring scale. Consensus (>70%) supported practical steps to strengthen monitoring: develop a short, validated tool for routine use; educate patients, families, and HCPs about CIAS; raise awareness via patient advocacy groups (PAGs); and deploy patient‑facing digital tools/apps to track cognitive symptoms.
